# Passively Administered Pooled Human Immunoglobulins Exert IL-10 Dependent Anti-Inflammatory Effects that Protect against Fatal HSV Encephalitis

**DOI:** 10.1371/journal.ppat.1002071

**Published:** 2011-06-02

**Authors:** Chandran Ramakrishna, Alain N. S. Newo, Yueh-Wei Shen, Edouard Cantin

**Affiliations:** 1 Division of Virology, Beckman Research Institute, City of Hope, Duarte, California, United States of America; 2 Immunology, Beckman Research Institute, City of Hope, Duarte, California, United States of America; 3 Neurology, Beckman Research Institute, City of Hope, Duarte, California, United States of America; Saint Louis University, United States of America

## Abstract

HSV-1 is the leading cause of sporadic encephalitis in humans. HSV infection of susceptible 129S6 mice results in fatal encephalitis (HSE) caused by massive inflammatory brainstem lesions comprising monocytes and neutrophils. During infection with pathogenic microorganisms or autoimmune disease, IgGs induce proinflammatory responses and recruit innate effector cells. In contrast, high dose intravenous immunoglobulins (IVIG) are an effective treatment for various autoimmune and inflammatory diseases because of potent anti-inflammatory effects stemming in part from sialylated IgGs (sIgG) present at 1–3% in IVIG. We investigated the ability of IVIG to prevent fatal HSE when given 24 h post infection. We discovered a novel anti-inflammatory pathway mediated by low-dose IVIG that protected 129S6 mice from fatal HSE by modulating CNS inflammation independently of HSV specific antibodies or sIgG. IVIG suppressed CNS infiltration by pathogenic CD11b^+^ Ly6C^high^ monocytes and inhibited their spontaneous degranulation *in vitro*. FcγRIIb expression was required for IVIG mediated suppression of CNS infiltration by CD45^+^ Ly6C^low^ monocytes but not for inhibiting development of Ly6C^high^ monocytes. IVIG increased accumulation of T cells in the CNS, and the non-sIgG fraction induced a dramatic expansion of FoxP3^+^ CD4^+^ T regulatory cells (Tregs) and FoxP3^−^ ICOS^+^ CD4^+^ T cells in peripheral lymphoid organs. Tregs purified from HSV infected IVIG treated, but not control, mice protected adoptively transferred mice from fatal HSE. IL-10, produced by the ICOS^+^ CD4^+^ T cells that accumulated in the CNS of IVIG treated, but not control mice, was essential for induction of protective anti-inflammatory responses. Our results significantly enhance understanding of IVIG's anti-inflammatory and immunomodulatory capabilities by revealing a novel sIgG independent anti-inflammatory pathway responsible for induction of regulatory T cells that secrete the immunosuppressive cytokine IL-10 and further reveal the therapeutic potential of IVIG for treating viral induced inflammatory diseases.

## Author Summary

We show that fatal HSV encephalitis (HSE) is caused by excessive brainstem inflammation. Once brainstem inflammation is initiated, antiviral drugs that inhibit only viral replication are ineffective in protecting against fatal HSE. Infusion of high doses of pooled human IgG (IVIG) is an effective anti-inflammatory treatment for various autoimmune diseases. One anti-inflammatory mechanism depends on sialylated IgGs (sIgG) present in limiting amounts (1–3%) in IVIG, hence the need for high doses of IVIG. We discovered a novel anti-inflammatory pathway mediated by low doses of IVIG independent of sIgG that prevented fatal HSE by suppressing CNS inflammation. The non-sIgG fraction of IVIG induced regulatory CD4^+^ T cells that produced the immunosuppressive cytokine IL-10 in the brainstem. Importantly, we show that IL-10 is critical for suppressing the generation of pathogenic inflammatory macrophages. Thus, IVIG has a remarkable ability to balance the host inflammatory responses to virus infection and thereby promotes virus clearance without bystander damage to the CNS, accounting for survival of all infected mice. Overall, our results provide important new insights in understanding IVIG's anti-inflammatory activity and further reveal its potential for use in treatment of viral inflammatory diseases.

## Introduction

Herpes simplex virus (HSV) is the leading cause of sporadic encephalitis, which, although rare, can be fatal or result in severe neurological deficits in survivors [1]. We reported previously that dysregulated CNS inflammatory responses cause fatal HSE in 129S6 (129) mice. Most importantly, we showed that once CNS inflammation was initiated by HSV entry into the brainstem, inhibiting virus replication could not prevent development of fatal HSE [2], [3]. Similar conclusions have emerged from studies with susceptible BALB/c mice [4].

IVIG comprises human polyclonal IgG derived from pooled plasma collected from thousands of healthy donors. Initially it was used to provide normal levels of circulating IgG as replacement therapy for primary and secondary immunodeficiencies [5], [6]. IVIG has a broad repertoire of neutralizing antibodies for various pathogens and neutralization is commonly assumed to be the mechanism of protection in secondary immunodeficiencies [7]. Remarkably, early reports that IVIG was able to prevent fatal HSE in BALB/c mice independently of neutralizing activity, even when administered up to 48 h post infection (pi), were not further investigated to elucidate the mechanism(s) of protection [8], [9].

IVIG is a FDA approved treatment for immune thrombocytopenia (ITP) and Kawasaki's vasculitis, and dramatic response rates that exceed 80% have been observed for ITP. The use of IVIG for treating a variety of autoimmune and systemic inflammatory diseases has steadily increased to include not only antibody mediated diseases, but also disorders caused by dysregulated cellular immunity, such as multiple sclerosis (MS), myasthenia gravis and graft versus host disease [10], [11]. IVIG has been reported to prevent development of experimental autoimmune encephalitis (EAE), an animal model of MS, by increasing both the frequency and suppressive activity of CD4^+^ T regulatory cells (Tregs) [12], [13]. Nonetheless, despite intense study, IVIG's mechanism(s) of action remain enigmatic, as discussed in several recent reviews [11], [14], [15], [16], [17], [18].

Based on studies in mouse models of ITP, serum induced arthritis and nephrotoxic nephritis, Ravetch and colleagues proposed a model to explain the sustained anti-inflammatory effects of IVIG. They proposed that IVIG interacted initially with a CSF-1 dependent ‘sensor’ cell, identified as a regulatory macrophage. The ensuing upregulation of the inhibitory FcγRIIb concomitant with down regulation of the activating FcγRIV has the net effect of raising the activation threshold of effector monocytes, thereby diminishing inflammation [16], [19]. An important recent finding by this group was that a subset of IgG molecules sialylated on Asp297 in the Fc domain could recapitulate the protective effects of IVIG in the ITP, nephrotoxic nephritis and arthritis models [20]. Sialylated IgGs (sIgG), which comprise 1–5% of IVIG, were effective at ∼10 fold lower concentration, which explains the requirement for high dose (1–2 g/kg) IVIG [16], [21].

We report here that low dose (150 mg/kg) IVIG protected all 129 mice from fatal HSE. IVIG mediated protection against HSE depended on its immunomodulatory activity but was independent of HSV specific antibodies. We confirmed the anti-inflammatory activity of purified sIgG, which is accessed with high dose IVIG. Importantly, we showed that the non-sIgG fraction of IVIG, corresponding to low dose IVIG, mediated equally potent anti-inflammatory effects to prevent fatal HSE. IVIG devoid of sIgG induced a dramatic expansion of Tregs and ICOS^+^ CD4^+^ T cells. Protection against fatal HSE was critically dependent on IL-10 produced primarily by the ICOS^+^ CD4^+^ T cells that accumulated in the CNS of IVIG treated but not PBS treated control mice. Although, signaling via the inhibitory FcγRIIb contributed to IVIG induced suppression of CNS infiltration, the absence of FcγRIIb did not abrogate protection against fatal HSE, which is characterized by accumulation of pathogenic Ly6C^high^ macrophages.

## Results

### A Single Dose of IVIG Is Sufficient to Protect 129 mice

**Figure 1:**
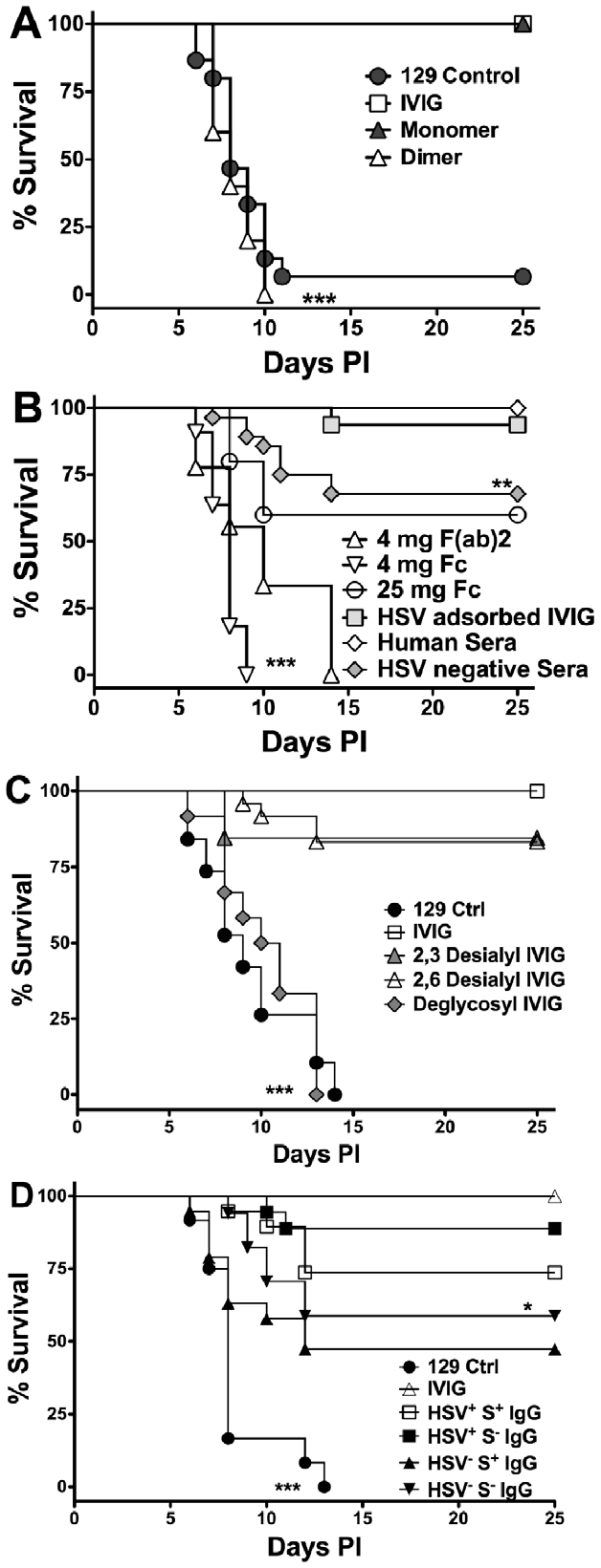
Protection against fatal HSE by IVIG and derivatives. HSV infected 129 mice were injected i.p. at 24 h pi with **(A)** PBS, 3.75 mg of IVIG, or monomeric or dimeric fractions of IVIG; **(B)** 3.75 mg of pooled HSV positive or negative sera, HSV adsorbed IVIG, 4 mg of F(ab)_2_, or 4 or 25 mg of Fc fragments; **(C)** 3.75 mg of IVIG, α2,3- or α2,6- desialylated IVIG or deglycosylated IVIG or PBS; **(D)** 1 mg of sialylated (S^+^) or 3.75 mg of non-sialylated (S^−^) IgG isolated from IVIG (HSV^+^) or HSV seronegative (HSV^−^) pooled sera and monitored for survival. Data are representative of 3–5 experiments (n = 16–50). **P<0.01 for 1B: HSV^+^ IVIG vs. HSV^−^ IVIG; *P<0.05 for 1D: HSV^+^S^−^ vs. HSV^−^S^−^ IgG; ***P<0.0001 for 1A: PBS and dimeric IgG vs. IVIG and monomeric IgG, 1B: 4 mg F(ab)_2_ or Fc fragments vs. IVIG or HSV adsorbed IVIG and 1C: IVIG vs. deglycosylated IVIG.

We investigated IVIG protection in a mouse model of HSE characterized by massive accumulation of macrophages and neutrophils in inflammatory lesions in the brainstem (BS) [3] using a dose of 3.75 mg IVIG/mouse, which was previously reported to protect all HSV infected BALB/c mice [9]. HSV inoculated 129 mice were injected i.p. with 3.75 mg IVIG 24 h pi and survival was compared to PBS treated control mice. While >90% of the control mice succumbed to HSE by 7–12 pi, all IVIG recipients survived (**Figure 1A**). Protection against fatal HSE depended on the timing of IVIG administration [9]. Although, IVIG prolonged survival of infected 129 mice when given at 72 or 96 h pi, it nonetheless failed to prevent mortality (**Figure S1**). Because IVIG preparations typically contain <1% aggregates and 3–15% IgG-dimers [22], [23] we evaluated the contribution of IgG-dimers and monomers to protection. When HPLC purified IVIG fractions were injected into mice 24 h pi, the monomeric, but not the dimeric, IgG fraction protected all mice from fatal HSE (**Figure 1A**). Hence, IVIG protection resides exclusively within the monomeric IgG fraction.

### HSV Neutralizing Activity Is Dispensable for IVIG Protection

Purified F(ab)_2_ and Fc fragments were injected into infected mice 24 h pi and the mice were monitored for survival. Neither the F(ab)_2_ or Fc fragment preparations were protective when given at 4 mg/mouse. However, increasing the Fc fragment dose to 25 mg/mouse (equivalent to high dose IVIG), protected ∼65% of mice (**Figure 1B**). IVIG is rich in neutralizing antibodies specific for HSV-1 and 2 [24], and that the F(ab)_2_ fragments retained neutralizing activity yet failed to protect suggested that neutralization was dispensable for IVIG mediated protection [8]. To demonstrate conclusively that IVIG protection against HSE was independent of HSV neutralizing activity, infected mice were given IVIG devoid of neutralizing antibodies 24 h pi. As expected, IVIG adsorbed free of neutralizing, but not non-neutralizing antibodies, was still able to protect against fatal HSE (**Figure 1B** **and** **Table 1**). To determine a role for non-neutralizing antibodies, pooled sera collected from donors seronegative for both HSV-1 and HSV-2 was administered to infected mice 24 h pi. Although, the absence of HSV specific antibodies in seronegative IVIG reduced protection only slightly relative to IVIG, the effect was statistically significant (**Figure 1B**, 70% versus 100%, p = 0.014), which suggests that non-neutralizing HSV specific antibodies present in the HSV adsorbed sera have a minor role in protection.

**Table 1:** Specificities of IVIG derivatives.

IVIG & Derivatives	HSV ELISA titer	Neutralizing Titer	Sialylation Titer
IVIG or Human Serum	1/10240	1/320	+ (1/625)
HSV Adsorbed IVIG	1/5120	<1/2	+ (1/625)
*DG – IVIG	1/5120	1/320	− (<1/5)
+α2,3 DS IVIG	1/5120	1/160	− (<1/5)
+α2,6 DS IVIG	1/5120	1/320	− (<1/5)
*HSV^+^ S^+^ IgG	1/5120	<1/2	+++
*HSV^+^ S^−^ IgG	1/5120	1/320	NA
HSV^−^ Pooled Sera	<1/5	<1/2	+ (1/1250)
⋆HSV^−^ S^+^ IgG	<1/5	<1/2	+++
⋆HSV^−^ S^−^ IgG	<1/5	<1/2	NA

*DG-IVIG, PNGase F digested IVIG to achieve deglycosylation.

**+:** DS-IVIG, Neuraminidase treated IVIG to remove terminal sialic acid residues at α2,3- or α2,6- position.

*^/⋆^S^+/−^ IgG, SNA lectin column eluted IgG to separate sialylated (S^+^) or non-sialylated (S^−^) from either IVIG or HSV negative sera.

NA, not applicable.

### Definition of the Protective Component(s) in IVIG

HSV infected mice were given deglycosylated IVIG or IVIG desialylated at either α2,3 or both the α2,3 and α2,6 linkages 24 h pi to determine the contribution of glycosylation, and more specifically sialylation, of IgG to protection in the HSE model. Deglycosylated IVIG failed to protect, consistent with glycosylation being mandatory for maintaining the functional integrity of the Fc domain, which is critical for protection by IVIG [25]. In contrast to the ITP and arthritis models, ≥80% of HSV infected mice treated with 3.75 mg IVIG desialylated with either α2,3 or α2,6 specific neuraminidases survived (**Figure 1C**), despite complete desialylation (**Figure S2**). To determine if sIgG (S^+^ IgG) could prevent HSE induced mortality, mice were given sIgG purified on *Sambucus nigra* (SNA) lectin affinity columns. Notably, >75% of infected mice given 1 mg sIgG purified from IVIG (HSV^+^S^+^ IgG) survived compared to ∼45% of mice given sIgG purified from pooled seronegative sera (HSV^−^S^+^ IgG) (**Figure 1D**). Protection declined with lower doses and a sIgG dose <0.5 mg failed to protect. Thus, sIgG can protect against fatal HSE when given at doses corresponding to high dose IVIG, much greater than that present in 3.75 mg IVIG. The non-sIgG (S^−^) fraction of IVIG also conferred statistically greater protection than that isolated from HSV seronegative IVIG when administered at 3.75 mg/mouse; >90% and ∼60% of mice survived, respectively (**Figure 1D**). Cumulatively, these results reveal a novel potent sIgG independent anti-inflammatory pathway mediated by low dose IVIG.

### IVIG Diminishes CNS Inflammation and Prolongs Integrity of the Blood Brain Barrier

**Figure 2:**
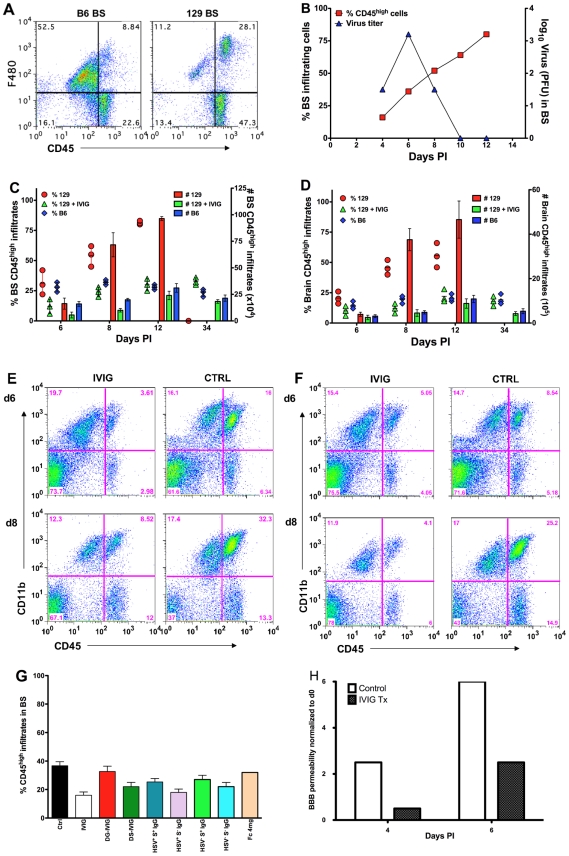
IVIG inhibits CNS inflammation in HSV infected mice. **(A)** Macrophages infiltrating the brainstem (BS) of infected B6 and 129 mice at d 12 pi. **(B)** CD45^high^ infiltrates (y-axis, left) and HSV titers (y-axis, right) in BS of 129 mice. Cells isolated from **(C)** BS or **(D)** brain of PBS or IVIG treated 129 and B6 mice were analyzed for CD45^high^ infiltrates at indicated times pi; left y-axis: % CD45^high^ infiltrates in BS shown as symbols; right y-axis: total number of CD45^high^ infiltrating cells as vertical bars. Data representative of 3 experiments (4 mice/time point). Error bars depict standard errors of mean. Representative FACS plots of **(E)** BS and **(F)** brain from IVIG- (left column) and PBS (right column) treated (right column) 129 mice. **(G)** Percentage of CD45^high^ infiltrates in BS of 129 mice treated with derivatives of IVIG or HSV seronegative sera at d6 pi. **(H)** Assessment of BBB permeability by sodium fluorescein uptake in infected 129 mice given PBS or IVIG.

Massive CNS inflammation is the primary cause of fatal HSE in 129 mice, while C57B6 mice, which exhibit minimal CNS inflammation, are resistant to HSE [3]. Flow cytometric analysis of leukocyte CD45^high^ infiltrates in the BS revealed that 129 mice had 75% CD45^high^ infiltrates compared to 30% CD45^high^ infiltrates for B6 mice at d12 pi (**Figure 2A**). Although 129 mice cleared infectious virus from the BS and trigeminal ganglia (not shown) by d10 pi, they nonetheless failed to control CNS inflammation (**Figure 2B**). Compared to control infected 129 mice, IVIG treated mice exhibited a dramatic reduction of infiltrating CD45^high^ peripheral leukocytes at d6, 8 and 12 pi (**Figure 2C–F**). At d6 pi, CD45^high^ infiltrates comprised ∼12% (range 6–18%) of total cells recovered from the BS of IVIG treated 129 mice compared to ∼30% (range 22–42%) in control 129 mice (**Figure 2C, E**). By d8 pi, CD45^high^ infiltrates comprised more than 55% (range 45–62%) of total BS cells in control mice compared to ∼24% (range 20–28%) in IVIG treated mice (**Figure 2C, E**). The majority of cells that infiltrated the BS of control 129 mice were CD11b^+^ macrophages and neutrophils (**Figure 2E**). The few surviving control mice exhibited even more pronounced inflammation (∼75%) at d12 pi (**Figure 2A, C**) compared to protected IVIG treated mice (∼30%, **Figure 2C**). When presented as total cell numbers the striking difference in leukocyte infiltration is even more dramatic, with an initial 3-fold difference in total CD45^high^ cells at d6 pi (5.5±2.6×10^4^ in IVIG treated mice compared to 1.6±0.5×10^5^ in controls) that escalated to a massive 7-fold difference by d8 pi (1±0.2×10^5^ in IVIG vs. 7.2±1.1×10^5^ in controls; **Figure 2C**). The reduced CNS inflammation in IVIG treated 129 mice mirrored the leukocyte CNS infiltration observed in untreated resistant B6 mice that survive (**Figure 2A, C–F**). It is also evident that IVIG's anti-inflammatory effects persist long-term (**Figure 2C**).

Leukocytes infiltrated the brain (**Figure 2D, F**) and spinal cord (**Figure S3**) of control mice robustly by d6 pi, and by d8 pi there was a 10-fold increase in infiltrates within the brains of control mice (3.8±2×10^5^ at d6 to 3.7±2.3×10^6^ at d8) compared to only a marginal increase in total CD45^high^ infiltrates in IVIG treated mice (2.5±1×10^5^ at d6 to 4.5±2×10^5^ total infiltrates at d8). Thus, IVIG regulates inflammation by diminishing the infiltration of cells into the CNS of infected mice. Impaired anti-inflammatory activity was responsible for the failure of deglycosylated IVIG to protect 129 mice (**Figure 1C**), as mice treated with 3.75 mg deglycosylated IVIG or Fc fragments had increased levels of CD45^high^ infiltrating cells, similar to control infected 129 mice (**Figure 2G**). We infer that the anti-inflammatory activity of sialylated Fc fragments accounted for survival of mice treated with 25 mg Fc fragments. Mice that were protected by treatment with desialylated IVIG, 3.75 mg of S^−^ IgG or 1 mg S^+^ IgG isolated from either IVIG or HSV seronegative IVIG also had reduced levels of CD45^high^ infiltrating cells, similar to IVIG treated mice (**Figure 2G**).

Vigorous inflammation in the CNS of 129 mice suggested the integrity of the blood brain barrier (BBB) might be severely compromised in infected control 129 mice but not in the IVIG recipients. Sodium fluorescein uptake assays performed to assess BBB integrity showed a >6-fold increase in uptake from d0 to 6 pi in BS of control mice in contrast to only a ∼2.5-fold increase for IVIG treated 129 mice (**Figure 2H and 2C–D**), consistent with the dramatic reduction in CNS inflammation in IVIG treated compared to control 129 mice. Notably, IVIG was excluded from the CNS, at least in the first 72 h post infusion, as determined by micro-PET imaging and biodistribution of Cu^64^-labeled IgG (**Figure S4**); hence, IVIG acted peripherally to modulate CNS inflammation.

### IVIG Alters the Composition of Leukocytes Infiltrating the CNS

**Figure 3:**
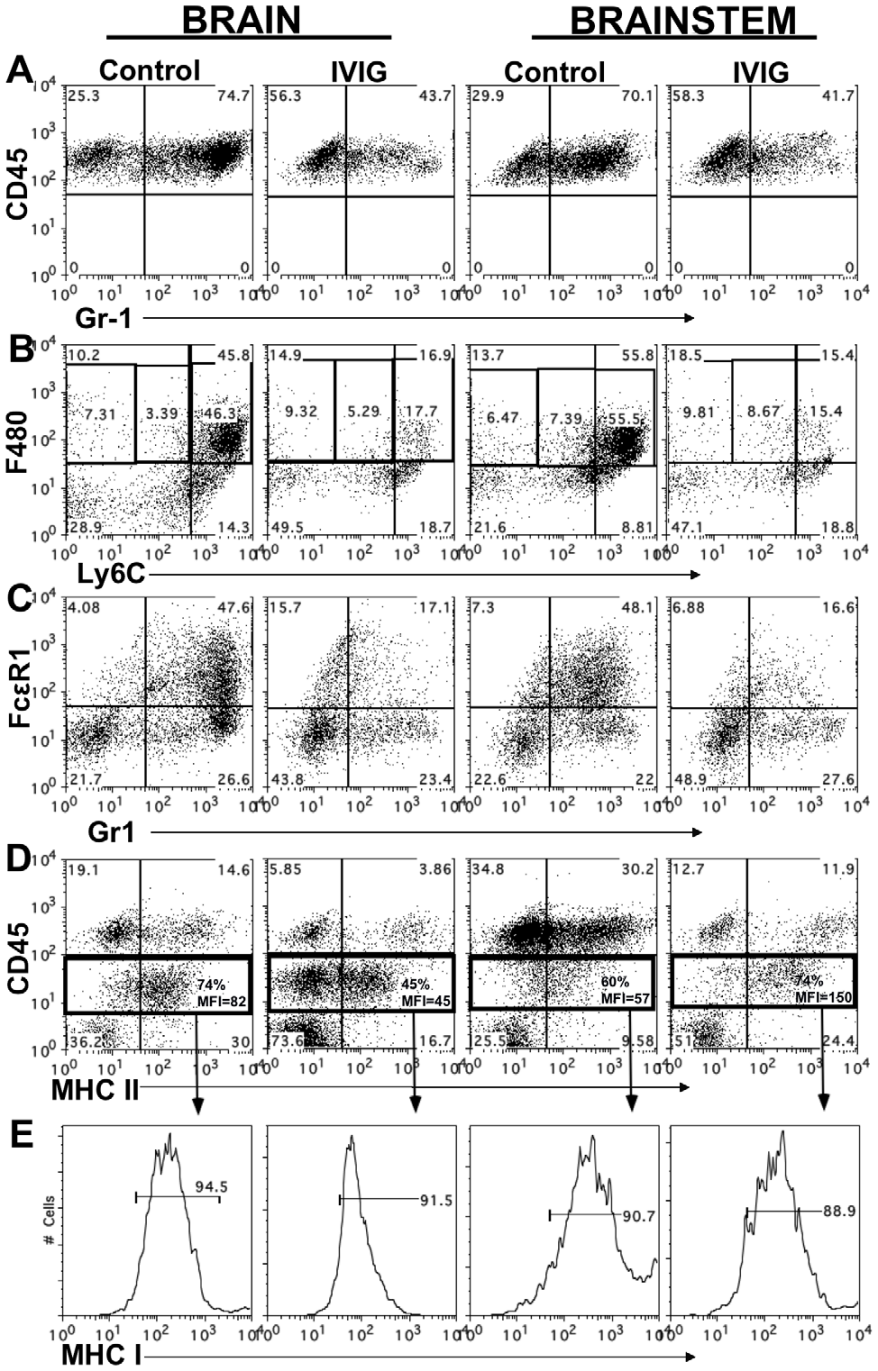
IVIG alters the phenotype of macrophages and microglia. CD45^high^ infiltrates in brain and BS of control or IVIG treated 129 mice at d8 pi analyzed for **(A)** Gr-1^+^ cells; **(B)** Ly6C^high^ and **(C)** FcεR1^+^ F480^+^ macrophages. **(D)** MHC II expression on CD45^high^ macrophages and CD45^int^ microglia isolated from BS or brains; frequencies and mean fluorescence intensity (MFI) of MHC II^+^ CD45 microglia (boxed regions) shown. **(E)** MHC I expression by CD45^int^ microglia. Data representative of 2–3 experiments (n = 6–12 mice).

At both d6 and 8 pi, >70% of the CD45^high^ leukocytes infiltrating the BS and brain of infected control 129 mice were Gr-1^+^ (**Table 2** and **Figure 3A**). IVIG treatment of infected mice dramatically reduced infiltration of this population to ∼40% of total CD45^high^ cells at d8 pi, equivalent to a ∼20-fold reduction in total Gr-1^+^ cells (**Figure 3A**, **Table 2**). Gr-1^+^ cells are comprised primarily of Ly6G^+^ CD11b^+^ neutrophils and Ly6C^high/int^ F480^+^ macrophages. Further analysis of the Gr-1^+^ cells in BS of IVIG treated mice revealed that although IVIG treatment reduced total numbers of Ly6G^+^ F480^−^ SSC^high^ neutrophils within this population (**Table 2**), the most dramatic decline was observed within the Ly6C^high^ inflammatory macrophage subset (**Figure 3B**). Ly6C^high^ F480^+^ SSC ^low^ macrophages, which comprised 50–60% of total CD45^high^ cells in both BS and brains of control mice, were reduced to ∼15–17% in the IVIG treated group (**Figure 3B**). Total percentages of F480^+^ macrophages were reduced from 66% in BS and 57% in brains of control mice to 28% and 30% in BS and brains, respectively, of IVIG treated mice, which constituted a 15-fold drop in total macrophage numbers in the CNS at d8 pi (**Figure 3B** **and** **Table 2**). The majority of Ly6C^high^ macrophages isolated from BS of control mice expressed high levels of FcεR1 as compared to markedly diminished expression on the Gr-1^+^ subset present in the BS of IVIG treated mice (**Figure 3C**). Thus, these data show early involvement of inflammatory Ly6C^high^ macrophages in the disease process of HSE, confirming earlier results, which showed that anti-Gr-1 mAb mediated depletion of macrophages and neutrophils prolonged survival of 129 mice [3], [26]. In addition to reducing the numbers of Ly6C^high^ macrophages, IVIG treatment altered the composition of BS infiltrating cells: IVIG treatment reduced infiltration of macrophages and neutrophils that predominated the CNS of control 129 mice while it increased T cell infiltration. Whereas only 10% and 20% of brain and BS infiltrates were composed of T cells in control mice, T cells comprised 40% and 55% of the brain and BS infiltrates of IVIG treated mice, respectively (**Table 2**). These results emphasize IVIG's capacity to regulate not only the generation of pathogenic macrophages, but also to control infiltration of different immune cell subsets into the CNS of HSV infected mice.

**Table 2:** IVIG reduces and alters infiltrating cell composition.

Cell subsets: BS of 129 mice	PBS: d6 pi	IVIG: d6 pi	PBS: d8 pi	IVIG: d8 pi
Total # in BS (x10^4^)^a^	71^a^	47	133	62
CD45^high^ infiltrates (% BS)^b^	26^b^	13	60	26
Gr-1 reactive cells^c^	78^c^	65	70	40
CD45^high^ CD11b^+^ F480^+^ Macs (MHC II^+^)^d^	55 (62%)^d^	40 (70%)^d^	65 (90%)^d^	30 (100%)^d^
Ly6G^+^ Neutrophils^c^	20	5	15	5
CD11c^+^ (CD11b^+^) cells^c^	30	12	18	15
T cells^c^	10	40	20	55
CD45^int^ microglia (MHC II^+^)^d^	18 (65%)^d^	15 (65%)	15 (65%)	20 (72%)

a Total numbers in BS represented as cells present in BS of indicated groups.

b CD45^high^ infiltrates are shown as percentage within BS mononuclear cells.

c All individual subsets are represented as percentages within CD45^high^ infiltrating cells.

d Numbers in parenthesis represent % MHC II^+^ cells within macrophage or microglial subsets.

### IVIG Modulates Activation of Macrophages and Microglia in the CNS

We analyzed MHC class I and II expression on microglia and macrophages by flow cytometry to determine their activation status. Although microglia in both control and IVIG treated mice expressed MHC class I at d6 pi, MHC class II expression was very low (mean fluorescence intensity [MFI]  = 0, **Table 2**). However, by d8 pi, MHC class II expression on microglia was greatly increased in the brain of control mice compared to IVIG treated mice (74% vs. 45%) (**Figure 3D**), and MHC class I expression also increased in brains and BS of both groups of mice (**Figure 3E**). Surprisingly, both the fraction of microglia that expressed MHC class II and the MFI were increased in the BS of IVIG treated mice as compared to control mice (74% vs. 60%, MFI 150 vs. 57; **Figure 3D**). Thus, IVIG treatment enhances the activation state of macrophages and microglia in the CNS, as demonstrated by MHC I/II (**Table 2**, **Figure 3**). Induction of MHC II expression on microglia in the CNS requires the presence of IFN-γ which is likely derived from T cells infiltrating the BS of IVIG treated mice (**Table 2**).

### IVIG Modulates Macrophage Degranulation

**Figure 4:**
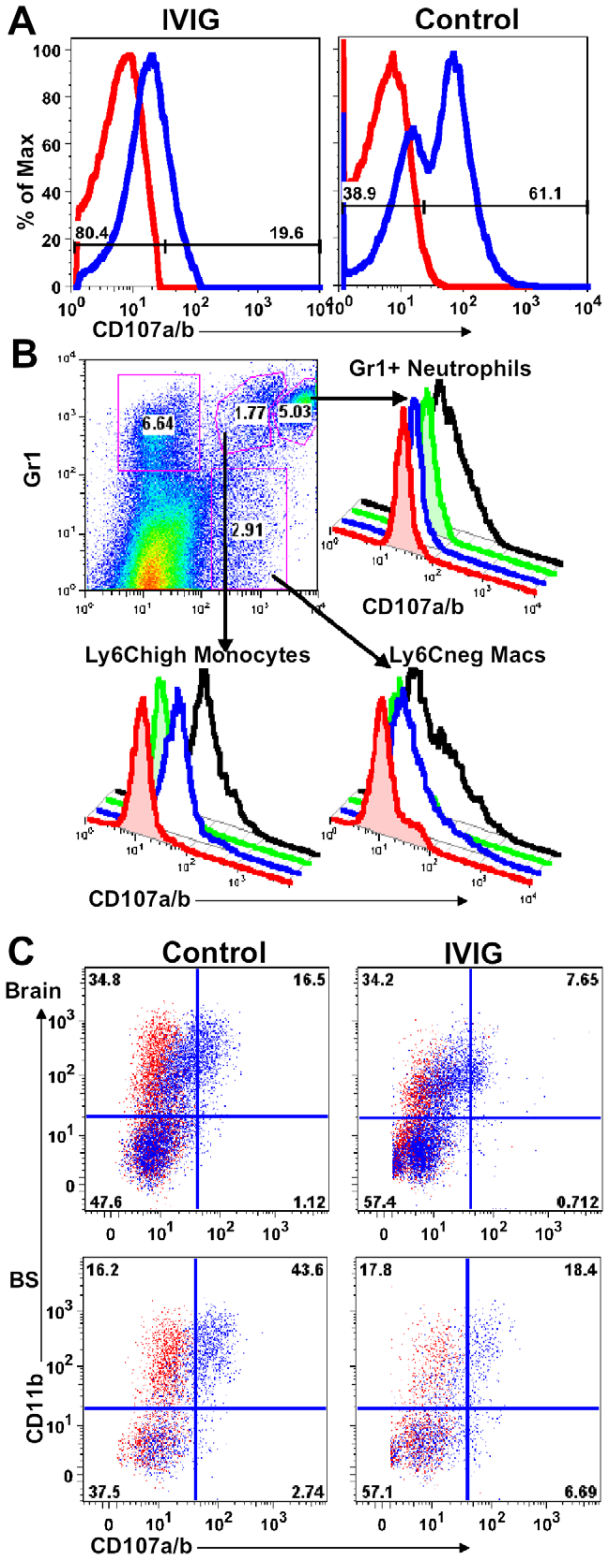
IVIG inhibits macrophage degranulation. **(A)** CD107a expression by splenocytes isolated at d6 pi from IVIG- (left plot) or PBS-treated (right plot) 129 mice. **(B)** Spleen cells isolated from PBS treated infected mice at d6 pi were delineated into three subsets based on CD11b and Gr-1 reactivity (top left plot). Gr-1^high^ CD11b^+^ neutrophils (top right), Gr-1^+^ CD11b^+^ inflammatory monocytes (bottom left) and Gr-1^−^ CD11b^+^ macrophages (bottom right) were analyzed for CD107a expression with (black lines) or without (blue line) incubation with heat-killed HSV. Isotype controls (red, without; green, with HK-HSV stimulation) are included. **(C)** Cells from brain (top) and BS (bottom) of PBS (left) and IVIG treated (right) 129 mice at d8 pi were analyzed for CD107a/b expression in the absence of antigen stimulation. Data representative of 2 experiments (n = 6–8 mice).

To determine if macrophages in peripheral lymphoid tissues have a different activation or degranulation profile, splenocytes obtained from control and IVIG treated HSV infected mice at d6 pi were stimulated *ex vivo* for 4 h with or without heat killed HSV (HK-HSV) antigen in the presence of antibodies to CD107a/b (LAMP-1 and 2). The majority of splenic macrophages isolated from control infected mice degranulated spontaneously and expressed high levels of surface CD107a, even in the absence of HK-HSV stimulation (**Figure 4A**), whereas, strikingly, those isolated from IVIG treated mice did not (**Figure 4A**). To identify the highly activated cell subset(s) in spleens of infected 129 mice with this phenotype, cells were phenotypically distinguished by surface expression of Gr-1, F480 and Ly6C. Both the Gr-1^+^ CD11b^+^ F480^−^ SSC^high^ neutrophils and the mature non-inflammatory macrophages (Ly6C^−^ F480^+^) degranulated only in response to stimulation with HK-HSV antigen, whereas the Ly6C^high^ inflammatory macrophages expressed high levels of CD107a without stimulation (**Figure 4B**). Similarly, there were more spontaneously degranulating CD45^high^ CD11b^+^ macrophages in the brain (32% vs. 16%) and BS (74% vs. 51%) of control mice compared to IVIG treated mice (**Figure 4C**). Moreover, as macrophages were more prevalent in infected BS of control PBS treated mice than in IVIG treated mice, the total numbers of degranulating macrophages were greatly increased (**Table 2** **and** **Figure 3**). Thus, macrophages in the CNS and lymphoid organs of IVIG recipients are anti-inflammatory, while those in control mice have a pathogenic phenotype.

### IVIG Alters FcR Expression on Macrophages and Monocytes

**Figure 5:**
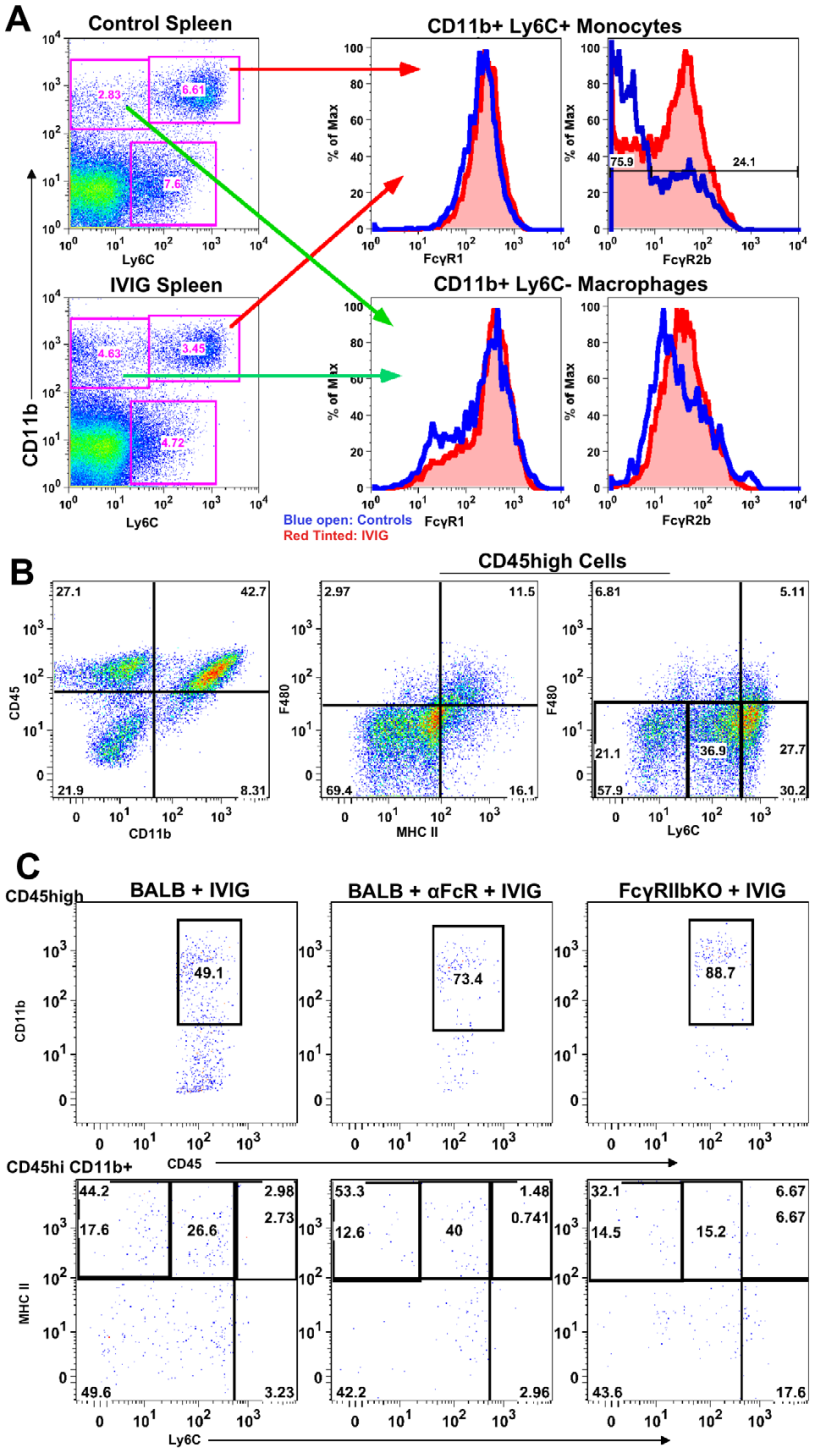
FcγRIIb limits CNS infiltration. **(A)** Ly6C^+^ (top right 2 plots) and Ly6C^−^ (bottom right 2 plots) subsets of CD11b^+^ splenic macrophages in control PBS (top left) and IVIG treated (bottom left) 129 mice at d 6 pi analyzed for FcγRI (middle plot) or FcγRIIb/III (right plot) expression. (blue lines: PBS treated macrophages; red lines: IVIG treated macrophages). **(B)** CD45^high^ CD11b^+^ monocytes in BS of anti-FcγRIIb/III mAb depleted IVIG treated 129 mice at d8 pi (left). F480^+^ macrophages analyzed for MHC II^+^ (middle) and Ly6C expression (right); plots gated for CD45^high^ infiltrates. **(C)** Infected BALB/c mice given IVIG following treatment with either isotype (left) or anti-FcγRIIb/III mAb (middle) and analyzed for CD45^high^ monocytes at d6 pi (top row); cells gated on CD45^high^ infiltrates. Infected FcγRIIb KO mice given IVIG and analyzed for infiltrating monocytes (right). CD45^high^ CD11b^+^ gated monocytes from these groups analyzed for Ly6C and MHC II^+^ expression (bottom row). Data representative of 2 experiments (n = 6–8 mice).

Modulation of FcR expression is one of many mechanisms proposed to explain IVIG's anti-inflammatory activity [27], [28]. Therefore, we examined FcR expression on monocytes from lymphoid organs of control and IVIG treated mice. Surface Ly6C^high^ expression facilitated discrimination of inflammatory from non-inflammatory CD11b^+^ F480^+^ macrophages (**Figure 5A**). Splenic Ly6C^−^ macrophages (**Figure 5A**) in control and IVIG treated mice expressed similar levels of the inhibitory FcγRIIb and activating FcγRIII receptors, as determined by CD16/32 reactivity. In contrast, the Ly6C^high^ inflammatory subset, which was more prevalent in the spleens of control mice, expressed much lower levels of FcγRIIb/III compared to those in the spleens of IVIG treated mice. FcγRI expression on both subsets was similar in both groups (**Figure 5A**). To determine a role for FcγRIIb/III receptors in IVIG's anti-inflammatory effects, CNS inflammation was analyzed at d8 pi in IVIG treated infected mice that were also treated with the 2.4G2 blocking mAb that inhibits FcγRIIb/III signaling [29]. Flow cytometry analysis revealed that the absence of FcγRIIb/III signaling compromised IVIG's ability to suppress CNS infiltration. With intact FcγRIIb/III signaling, there were ∼20–25% CD45^high^ infiltrating cells in the CNS of IVIG treated mice (**Figure 2C and E**). However, inhibition of FcγRIIb/III signaling resulted in increased CNS infiltration with ∼70% CD45^high^ cells present in the BS of these mice (**Figure 5B**). Interestingly, in wild-type (WT) mice, IVIG reduced macrophage influx while it increased infiltration of T cells (**Table 2**), whereas in the absence of FcγR signaling, CD11b^+^ F480^−^ monocytes dominated BS infiltrates (**Figure 5B**). The majority of monocytes, however, expressed intermediate levels of Ly6C and low levels of MHC II (**Figure 5B**), indicating that they were not inflammatory. Thus, FcγR signaling is required for IVIG mediated suppression of CNS infiltration, but not for modulation of pathogenic inflammatory macrophages.

To determine whether the inhibitory FcγRIIb receptor or the activating FcγRIII receptor was critical for suppression of inflammation, we compared CNS inflammation in infected IVIG treated BALB/c FcγRIIb knock-out (KO) mice at d6 pi to that in IVIG treated BALB/c mice and IVIG treated BALB/c mice given anti-FcγRIIb/III antibodies. IVIG suppressed CNS leukocyte infiltration in BALB/c mice as effectively as in 129 mice, but it failed to suppress CNS infiltration in either FcγRIIb/III depleted or FcγRIIb KO mice at d6 and 8pi (**Table 3**). Similar to results seen with 129 mice, the absence of FcγRII/III signaling skewed CNS infiltrates to predominantly CD11b^+^ monocytes rather than T cells (**Table 2** and **Figure 5C**), but, very few of these monocytes were Ly6C^high^, indicating they were not inflammatory (**Figure 5C**). The results shown in **Table 3** indicate that signaling via FcγRIIb rather than FcγRIII contributed to IVIG mediated modulation of CNS infiltration and modulation of the composition of cellular infiltrates. Surprisingly, despite unmitigated CNS leukocyte infiltration in IVIG treated 129 and BALB/c mice deficient in FcγRIIb signaling, all mice survived and showed no symptoms of encephalitis.

**Table 3:** FcγRIIb is required for control of CNS infiltration.

Cell subsets: in BALB/c BS	PBS d6pi	IVIG d6 pi	αFcγRIIb/III mAb + IVIG d6 pi	FcγRIIb KO mice + IVIG d6 pi	PBS d8 pi	IVIG d8 pi	FcγRIIb KO mice + IVIG d8 pi
Total # in BS (x10^4^)^a^	60 a	30	50	40	170	75	120
CD45^high^ infiltrates	32^b^	20	40	36	76	40	66
CD45^hi^ CD11b^+^ cells^c^	70	49	74	89	82	40	60
T cells^c^	10	40	17	8	15	55	35

a Total numbers in BS represented as cells present in BS of indicated groups.

b CD45^high^ infiltrates are shown as percentage within BS mononuclear cells.

c All individual subsets are represented as percentages within CD45^high^ infiltrating cells.

### IVIG Induces Regulatory CD4^+^ T Cells that Control Hyper-Inflammatory Responses

**Figure 6:**
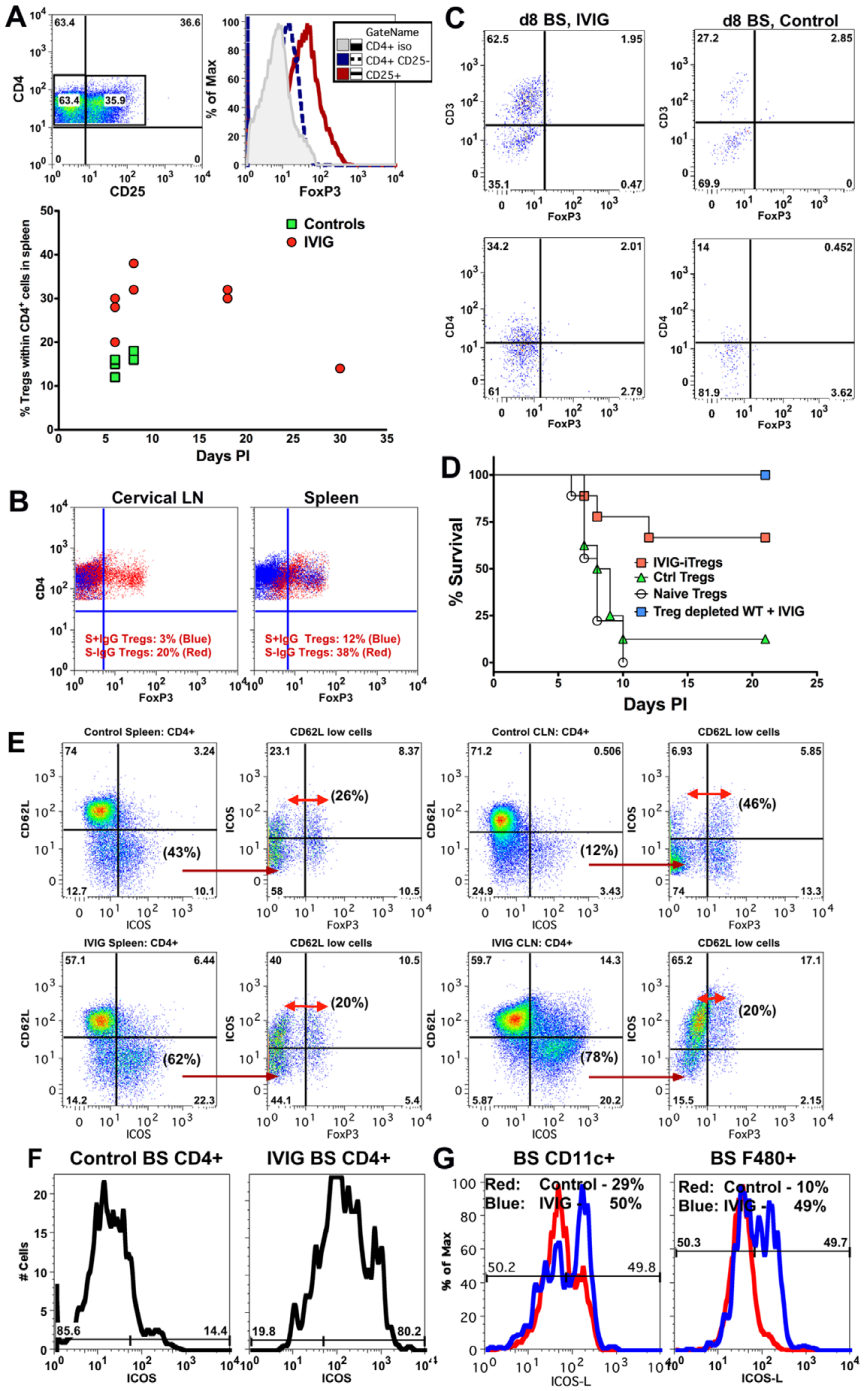
IVIG induced Tregs mediate but are not essential for anti-inflammatory effects in the CNS. **(A)** Representative FACS plots of CD4^+^ Tregs in the spleens of IVIG treated HSV infected 129 mice (top, left plot) expressing FoxP3 (top right). Percentage of FoxP3^+^ Tregs in CD4^+^ splenocytes of PBS or IVIG treated infected 129 FoxP3-GFP mice at the indicated time points pi (bottom). **(B)**, Percentage of Tregs within CD4^+^ T cells in CLN (left) and spleens (right) at d6 pi in infected 129 FoxP3-GFP mice given either S^−^IgG (red dots) or S^+^IgG (blue dots). **(C)** BS CD45^high^ infiltrates in IVIG (left) or PBS (right) treated (right) infected 129 FoxP3-GFP mice at d8 pi were analyzed for FoxP3^+^ CD3^+^ (top) or CD4^+^ (bottom) T cells; plots depict CD45^high^ gated cells. **(D)** Infected 129 mice given IVIG were depleted of Tregs using anti-CD25 mAb and monitored for survival (blue squares). FoxP3^+^ CD4^+^ Tregs isolated from the spleens of IVIG treated HSV induced (red square), PBS treated HSV induced (green triangles) or naïve mice (circles) were transferred into 129 WT mice that were infected with HSV and monitored for survival. **(E)** Representative FACS plots showing activated ICOS^±^ CD4^+^ T cells in spleens (left 2 plots) and CLN (right 2 plots) of PBS (top row) and IVIG (bottom row) treated (bottom row) HSV infected 129 FoxP3-GFP mice at d8 pi. 2^nd^ and 4^th^ plots gated on CD62L^low^ CD4^+^ T cells. **(F)** BS cells in PBS (left) or IVIG treated (right) HSV infected mice at d6 pi gated on CD45^high^ CD4^+^ T cells analyzed for ICOS expression. **(G)** BS CD45^high^ CD11c^+^ DCs (left) or F480^+^ macrophages (right) in PBS (red lines) or IVIG treated (blue lines) infected mice at d6 pi probed for ICOSL expression. Data representative of 2 experiments (n = 6–8 mice).

T cells were predominant in CNS infiltrates of IVIG treated mice at d8 pi. IVIG expanded CD4^+^ Tregs that contributed to protection in two different models of pathogenic inflammation [12], [30]. We used 129 FoxP3-GFP reporter mice to investigate the possibility that IVIG induced Tregs in the HSE model. Analysis of HSV infected, IVIG treated 129 FoxP3-GFP reporter mice revealed that the majority of the CD4^+^ T cells in the spleen were FoxP3^+^ CD25^+^ Tregs at d8 (∼40%) and d18 pi (∼30%, **Figure 6A**). In contrast, the percentage of Tregs was only modestly increased in the spleens of infected PBS treated control mice (∼15%, **Figure 6A**) and these Tregs were obviously ineffective in suppressing the exaggerated CNS inflammatory responses. To determine whether the S^+^ or S^−^ IgG component of IVIG induced Tregs, we analyzed 129 FoxP3-GFP reporter mice treated separately with the two preparations at d6 pi (**Figure 6B**). S^−^ IgG dramatically expanded Tregs in the cervical lymph nodes (CLN) (20%) and spleen (38%), while S^+^ IgG did not induce Tregs (3% in CLN and 12% in spleen). Interestingly, FoxP3^+^ CD4^+^ Tregs were not present in the infected BS of either IVIG treated or control mice, suggesting the Tregs function primarily in lymphoid organs (**Figure 6C**). We used adoptive transfer of IVIG induced or control Tregs from infected or naïve mice to assess the contribution of Tregs to IVIG's anti-inflammatory effects, which facilitated assessing their intrinsic suppressive potential. FoxP3^+^ GFP^+^ Tregs isolated from the spleens of naïve, IVIG and PBS treated HSV infected FoxP3-GFP mice on d8 pi were adoptively transferred into naive 129 recipients that were challenged 24 h later with HSV. Most recipients of Tregs isolated from IVIG treated HSV infected FoxP3-GFP mice, but not control PBS treated or naïve mice, were protected long-term (**Figure 6D**). This result shows that adoptively transferred IVIG induced Tregs prevented lethal HSE while Tregs from the PBS treated control or naïve mice were non-functional. We investigated the requirement for Tregs in protection afforded by IVIG by using anti-CD25 antibodies to deplete Tregs prior to HSV infection and during subsequent IVIG treatment. Unexpectedly, all Treg depleted mice survived HSV challenge, which indicated that Tregs were not essential for IVIG induced suppression of pathogenic CNS inflammatory responses (**Figure 6D**).

To determine if, in addition to Tregs, another T cell subset was involved in IVIG's anti-inflammatory effects, we analyzed spleen and CLN CD4^+^ T cells for expression of cell surface markers such as ICOS, GARP, CD103 and GITR that are characteristic of regulatory T cells. Only ICOS was dramatically up regulated on CD4^+^ T cells isolated from both CLNs and spleens of IVIG treated mice compared to control mice (**Figure 6E**). Upregulation of ICOS expression occurred preferentially within the activated CD62L^low^ population (spleen—IVIG: 62% vs. control: 43%; CLN—IVIG: 78% vs. control: 12%). Importantly, only ∼20–26% of the ICOS^+^ CD62L^low^ CD4^+^ T cells in the spleens of both groups of mice were FoxP3^+^ Tregs. Similarly, the majority of activated ICOS^+^ CD4^+^ T cells in the CLN of IVIG treated mice did not express FoxP3, indicating that they were not Tregs, while about 46% of the corresponding activated CD62L^low^ ICOS^+^ CD4^+^ T cells in control mice were FoxP3^+^ Tregs. Importantly, ICOS^+^ FoxP3^−^ CD4^+^ T cells were the major constituents of the CD4^+^ T cell population in the BS of IVIG treated mice but not of control mice at d6 pi (**Figure 6F**). Moreover, both CD11c^+^ DC and F480^+^ macrophages isolated from the BS of IVIG treated 129 mice at d6 pi expressed higher levels of ICOS-L, the ligand for ICOS, compared to those from control 129 mice (**Figure 6G**). These data show that IVIG elicits different populations of regulatory CD4^+^ T cells, and that whereas Tregs act primarily in peripheral lymphoid organs, ICOS^+^ CD4^+^ T cells may function both in the periphery and at sites of inflammation in the BS.

### IL-10 Is Critical for IVIG's Anti-Inflammatory Effects

**Figure 7:**
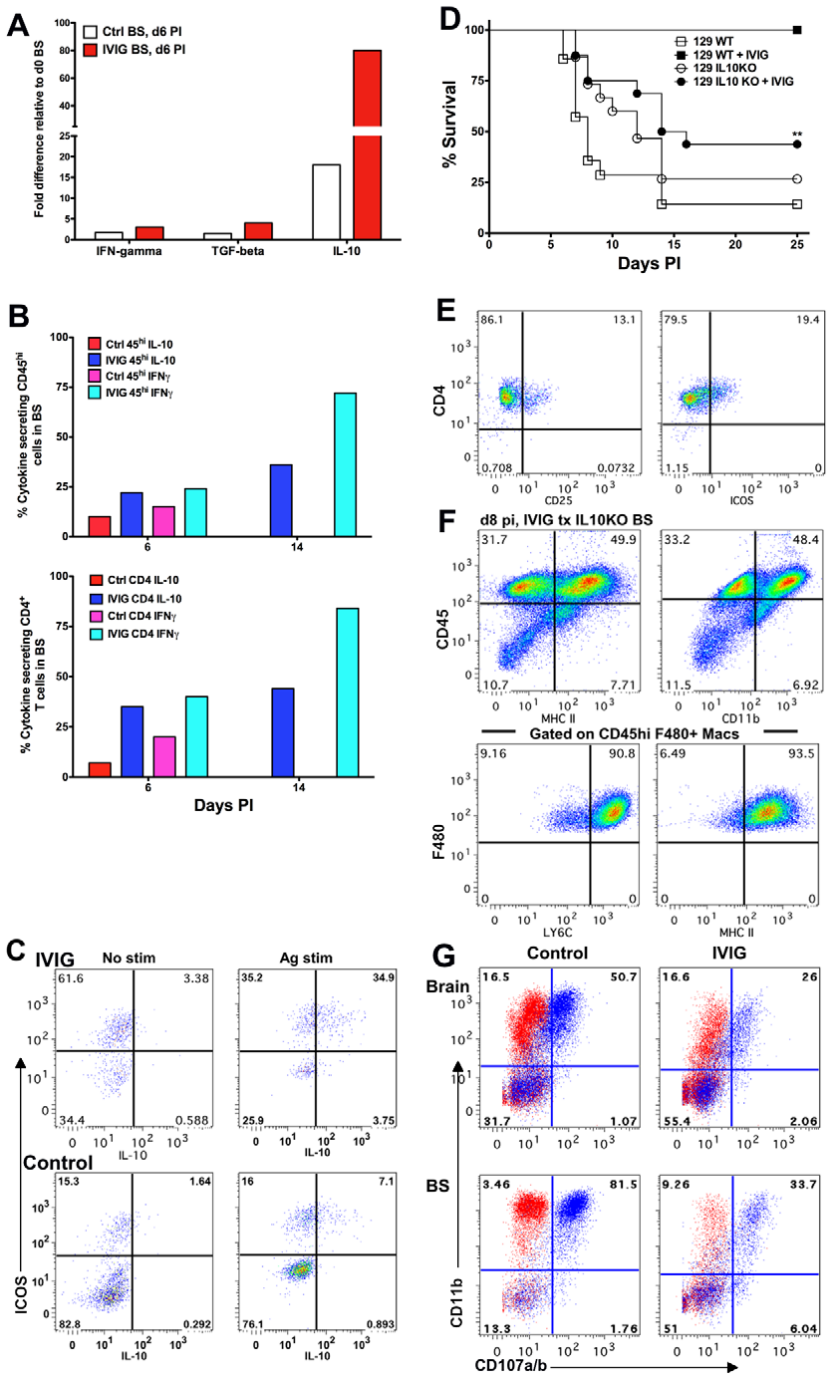
IL-10 is required for IVIG's anti-inflammatory effects in the CNS. **(A)** Quantification of IFN-γ, TGF-β and IL-10 mRNA at d6 pi in BS of infected 129 mice treated with PBS or IVIG. **(B)** Intracellular IFN-γ and IL-10 expression after antigen stimulation of CD45^high^ (top) or CD4^+^ T cells (bottom) at d6 and d14 pi in the BS of infected 129 mice given either IVIG or PBS. **(C)** BS CD4^+^ T cells in IVIG treated (top) or control (bottom) 129 mice at d6 pi analyzed for ICOS and IL-10 expression with (right) or without (left) PMA + ionomycin; plots gated on CD45^high^ CD4^+^ T cells. **(D)** HSV infected 129 WT or IL-10 KO mice were given IVIG or PBS at 24 h pi and monitored for survival. **(E)** FACS plots gated on CLN derived CD4^+^ T cells showing CD25 and ICOS expression. **(F)** BS CD45^high^ infiltrates in IVIG treated HSV infected IL-10 KO mice at d8 pi analyzed for expression of MHC II and CD11b (top). FACS plots gated on BS CD45^high^ F480^+^ macrophages depicting Ly6C and MHC II expression (bottom). **(G)** Degranulation of CD45^high^ gated cells as measured by CD107a/b expression (blue dots) in the absence of HK-HSV stimulation in the brain (top) and BS (bottom) of PBS (left) or IVIG (right) treated (right) infected IL-10 KO mice at d 8 pi. Isotype control shown in red. Data representative of 2–4 experiments (n = 6–12 mice).

To determine the mechanism(s) by which regulatory CD4^+^ T cells suppress inflammatory CNS responses, we used the *RT^2^*Profiler PCR array kit (SABiosciences, Frederick, MD) to compare the expression profiles of inflammatory genes in the BS of control and IVIG treated mice at d6 pi to those of uninfected mice. Expression of IFN-γ and TGF-β was moderately increased in BS of IVIG treated mice compared to uninfected mice, but not significantly compared to control PBS treated infected mice (**Figure 7A**). In contrast, relative to control uninfected mice, the BS of IVIG treated infected mice showed an ∼80-fold increase in IL-10 mRNA expression compared to only an 18-fold increase in BS of control PBS treated mice. Concordant with the RT-PCR results, intracellular flow cytometric analysis following stimulation with PMA and ionomycin revealed significantly increased IFN-γ and IL-10 expression by CD45^high^ cells, specifically the CD4^+^ T cells isolated from the BS of IVIG treated mice at d6 pi (**Figure 7B**, **S5**). Interestingly, both CD45^int^ microglia and a population of CD45^neg^ cells, possibly astrocytes, in the BS of both groups of mice secreted IL-10, even without stimulation (**Figure S5**). Furthermore, by d14 pi there was an even greater increase in the percentages of IFN-γ and IL-10 secreting CD45^high^ and CD4^+^ T cells in the BS of IVIG treated mice. Importantly, only the ICOS^+^ CD4^+^ T cells in both IVIG treated and control PBS treated mice secreted IL-10 following stimulation (**Figure 7C**). ICOS^+^ cells dominated the CD4^+^ T cell population in the BS of IVIG treated mice (70%) but not the BS of control mice (23%). IL-10 secreting cells comprised more than 50% of the ICOS^+^ CD4^+^ population in the BS of IVIG treated mice compared to ∼30% in the BS of control PBS treated mice (**Figure 7C**). These results suggest that ICOS and IL-10 may orchestrate suppression of hyper-inflammatory immune responses in the CNS of infected mice.

To determine whether IL-10 was critical for IVIG mediated protection against HSV induced CNS inflammation, we compared the outcome of infection in 129 WT and IL-10 KO mice treated with either IVIG or PBS at 24 h pi. As expected, the majority of 129 WT and IL-10 KO mice treated with PBS succumbed to HSE. While IVIG protected all 129 WT mice, only ∼45% of IVIG treated IL-10 KO mice survived to d25 pi (**Figure 7D**) and, of these mice, some exhibited symptoms of neurological sequelae, including altered gait, hunched back and weight loss.

Having established a critical role for IL-10 in IVIG protection against fatal HSE, it was important to show that impaired protection of IL-10 KO mice by IVIG was due to impaired suppression of CNS inflammation. Comparison of inflammatory responses in the BS of infected 129 IL-10 KO and WT mice treated with IVIG revealed that in the absence of IL-10, CNS inflammation continued unabated in the IL-10 KO mice (**Figure 7F** and **2E**). High levels of CD45^high^ infiltrates (82%) were present in the BS at d8 pi, the majority of which were inflammatory Ly6C^high^ MHC II^+^ CD11b^+^ F480^+^ macrophages (**Figure 7F**). Therefore, we concluded that IL-10 is essential for IVIG to suppress CNS inflammation and inhibit development of pathogenic Ly6C^high^ inflammatory macrophages. Interestingly, in the absence of IL-10, macrophages rather than T cells dominated immune cell infiltrates in the BS of IVIG treated 129 WT mice (**Figure 3B**, **7F** **and** **Table 2**). Concordant with their inflammatory phenotype, the majority of macrophages isolated from the brain and BS of PBS and IVIG treated IL-10 KO mice degranulated, even in the absence of HK-HSV, confirming their pathogenic phenotype (**Figure 7G**). Importantly, macrophages from IVIG treated IL-10 KO mice degranulated much more than macrophages isolated from IVIG treated 129 WT mice (62% vs. 16% for brain and 80% vs. 51% for BS, respectively) (**Figures 4C** **and** **7G**). This effect was even more exaggerated if the increased numbers of macrophages in the BS of IL-10 KO mice compared to WT mice was considered (**Figures 3** **and** **7**). To determine if CD4^+^ T cells were compromised in IL-10 KO mice, we compared expression of ICOS in peripheral lymphoid organs to that in 129 WT mice treated with IVIG. Expression of both CD25 and ICOS was reduced in the CLN of IL-10 KO mice compared to 129 WT mice (**Figure 7E**). Cumulatively, these results suggest that IL-10 secreting CD4^+^ ICOS^+^ T cells may drive suppression of inflammation and modulation of inflammatory macrophages. The potential regulatory role of IVIG induced ICOS^+^ CD4^+^ T cells in suppression of macrophage activation and CNS inflammation is currently under investigation.

## Discussion

We reported previously that HSE progression was not affected by Acyclovir inhibition of HSV replication after virus had entered the BS, which is consistent with immune mediated pathology rather than virus cytopathology causing death [3]. The number of clinical reports speculating on a role for immune pathology in HSE has also been increasing [31], indicating increased interest in this idea. In our studies of IVIG mediated protection against fatal HSE, 100% of 129 mice treated with low dose (3.75 mg/mouse) IVIG 24 h pi were protected from fatal HSE. This dose of IVIG is much lower than the 1–2 g/kg high dose typically used for treatment of autoimmune diseases [28], [32]. The monomeric IgG fraction of IVIG was protective, while the dimeric IgG fraction comprised of idiotype-anti-idiotype pairs [23], [33] was not. Purified Fc fragments have been shown to suppress ITP and Kawasaki disease in humans [18]. In our studies, at low dose (4 mg/mouse), Fc fragments failed to protect, whereas a high dose of Fc (25 mg/mouse) conferred significant protection, analogous to results obtained for ITP, nephrotoxic nephritis and serum transfer arthritis in experimental mouse models given 25 mg IVIG/mouse [28], [34].

Protection against fatal HSE by IVIG was independent of HSV specific antibodies and entry into the CNS during early acute infection. Rather, protection correlated with early and profound suppression of pathogenic inflammatory responses in the BS of infected IVIG treated 129 mice, which effectively prolonged the integrity of the BBB. In addition to markedly reducing CNS infiltration of CD45^high^ Ly6C^high^ inflammatory monocytes, IVIG treatment profoundly altered the phenotype of these cells. A particularly impressive manifestation of IVIG's potent immunomodulatory effects was the suppression of spontaneous and antigen induced degranulation by highly activated Ly6C^high^ monocyte/macrophages isolated from spleen, CLN and BS of HSV infected mice. Thus, IVIG acted peripherally to modulate early innate responses emanating from both infiltrating and resident immune cells in the CNS to protect against fatal HSE. We infer that failure of IVIG to protect when given at later times after infection is likely due to CNS infiltration by non-modulated aggressive Ly6C^high^ inflammatory monocytes prior to IVIG administration.

Ravetch and colleagues have provided extensive data to show that purified sIgG, which is present at 1–3% in IVIG, afforded complete protection against ITP and arthritis when given at a 10-fold lower dose than the usual high dose of 1.0 g/kg, thereby explaining the need for high dose IVIG in order to access its anti-inflammatory activity [21], [32]. They showed further that specific removal of α2,6 linked sialic acid residues on Asp297 in the Fc domain or deglycosylation, which impairs structural integrity of the Fc domain, both abolished IVIG's anti-inflammatory activity in ITP and arthritis models [21], [32], [35]. We speculated that the sIgG mechanism applied also to the HSE model. Deglycosylation of IVIG abrogated protection against fatal HSE, confirming the critical role of the Fc domain. In contrast, desialylation of IVIG did not significantly reduce protection against fatal HSE, contrary to results from the ITP and arthritis models [21], [32]. Notably, when HSV infected mice were given escalating doses of affinity purified sIgG ranging from 100 ng to 1 mg, robust protection against fatal HSE (∼70% survival) was observed only with doses >500 ng; drastically reduced survival was observed with doses less than 500 ng. Similar results were obtained with the purified Fc fragment. It was obvious from these results that sIgG could not be responsible for the protective effects of low dose IVIG, as it was present in too low an amount compared to the amount of purified sIgG required for robust protection. This important conclusion predicted that IVIG devoid of sIgG (HSV^+^S^−^ IgG) would be protective, and indeed, >90% of infected mice given 3.75 mg of HSV^+^S^−^ IgG survived. Furthermore, CNS inflammation in these mice was reduced to an extent comparable to that seen with IVIG or sIgG treated mice. Thus, we have identified a sIgG independent anti-inflammatory pathway mediated by low dose IVIG that is as potent as IVIG.

HSV^+^S^−^ IgG conferred statistically greater protection compared to HSV^−^S^−^ IgG, which is consistent with greater protection afforded by HSV seropositive compared to seronegative IVIG. Experimentally, immune complexes (ICs) formed *in vitro* or *in vivo* can mimic the protective effect of IVIG in, for example, ITP and serum induced arthritis models [16], [36], [37], [38]. Since IC formation would be expected in infected mice receiving HSV seropositive compared to seronegative IgG, we are investigating the potential role of ICs in mediating the anti-inflammatory effects of low dose IVIG.

Several studies reported an indispensable role for expression of the inhibitory FcγRIIb on effector macrophages and monocytes in IVIG's anti-inflammatory effects [39], [40], [41]. IVIG markedly upregulated FcγRIIb expression on CD11b^+^ Ly6C^high^ inflammatory monocytes in HSV infected mice, while expression of the activating FcγR1 was unaffected. Suppression of CNS infiltration was abolished by treating 129 and BALB/c mice with the 2.4G2 blocking mAb that targets FcγRIIb and FcγRIII or by genetic ablation of FcγRIIb signaling in BALB/c mice. Signaling via FcγRIIb in IVIG treated mice biased CNS infiltrates in favor of T cells rather than the predominant monocyte influx seen in PBS treated control mice. Remarkably, IVIG was still able to inhibit development of highly activated Ly6C^high^ inflammatory monocytes in BALB/c mice deficient in FcγRIIb expression, and all mice survived despite high levels of cells infiltrating the CNS. An important finding in the HSE model was that following IVIG treatment, FcγRIIb signaling acted primarily to limit CNS infiltrates and modulate their composition, but was dispensable for modulation of monocyte activation.

The observation of potent up regulation of IL-10 transcripts in the BS of IVIG treated infected mice revealed that IVIG induced functional Tregs. An important new finding was that the non-sIgG fraction of IVIG was primarily responsible for induction of Tregs. Regulatory T cell epitopes (Tregitopes) capable of inducing Tregs were recently identified in the Fc domain of IgG [42]. However, our results imply that IVIG induces Tregs independently of presentation of Tregitopes, since purified sIgG was unable to induce Tregs even though it should contain Tregitopes. IVIG has been reported to induce Tregs in various models of inflammatory disease [12], [43], which could account for its long-term protective effects, including in our HSE model. Using EGFP-FoxP3 reporter mice, we showed that IVIG significantly expanded FoxP3^+^ Tregs and that Tregs purified from infected IVIG treated, but not control PBS treated, EGFP-FoxP3 mice protected infected 129 recipients not treated with IVIG from fatal HSE. Thus, IVIG not only expands Tregs, but also augments their effector function. Unexpectedly, Treg depletion during infection of IVIG treated mice did not prevent anti-inflammatory responses or reduce survival. Possible explanations are that IVIG induced redundant regulatory cell populations, including, for instance, Tr1 regulatory T cells that secreted high levels of IL-10 [44], [45], [46]. We observed a dramatic expansion of ICOS^+^ FoxP3^−^ CD4^+^ T cells in the spleen and draining CLN of IVIG treated, but not control PBS treated, infected mice, and these cells also localized to the BS. ICOS is expressed by Tregs and plays a role in their regulation but it is also expressed on other regulatory cells, such as Tr1 cells [47], hence we are investigating the potential regulatory role of the ICOS^+^ CD4^+^ T cells induced by IVIG.

The immunosuppressive cytokine IL-10 was essential for protection against pathogenic hyper-inflammatory responses in the CNS, since survival of infected IVIG treated 129 IL-10 KO mice was drastically reduced. Notably, IVIG failed to suppress CNS infiltration or spontaneous degranulation by splenic macrophages from IL-10 KO mice. Highly activated inflammatory Ly6C^high^ macrophages were predominant in the extensive CD45^high^ infiltrates in BS of infected IL-10 KO mice. In contrast, IVIG treated FcγRIIb KO mice survived, despite high levels of CNS CD45^high^ infiltrates comparable to those in IL-10 KO mice, which implicates the Ly6C^high^ inflammatory macrophages as being causally involved in the high mortality of IL-10 KO mice. Thus, these results emphasize that the quality of the inflammatory response rather than just its magnitude is the critical determinant of pathogenic outcome.

Significant induction of IL-10 and IFN-γ expression by IVIG in primarily CD4^+^ T cells was evident early after treatment in the BS. Interestingly, ICOS^+^ CD4^+^ T cells that were predominant amongst CD4^+^ T cells in BS of IVIG treated, but not control, mice were the major producers of IL-10. HSV infection of microglia, even though abortive, has been reported to induce robust expression of proinflammatory cytokines and chemokines. *In vitro*, exogenous IL-10 was shown to attenuate this response [48], [49], [50]. IL-10 can exert autocrine inhibitory effects on macrophages and dendritic (DCs) cells that inhibit development of T_H_1- and T_H_2-type responses [51], while IL-10 produced by T_H_1, T_H_2 and T_H_17 cells represents a feedback loop to regulate the effector functions of macrophages and DCs [52]. These important immunoregulatory functions of IL-10 fit well with its essential role in promoting balanced inflammatory responses in IVIG treated mice that favor clearance of HSV without bystander immune pathology. Cumulatively, these results show that IVIG induced IL-10 producing ICOS^+^ CD4^+^ T cells are required for long-term regulation of CNS inflammatory responses.

One model to explain IVIG's anti-inflammatory activity proposes that sIgGs present at <3% in IVIG trigger anti-inflammatory responses upon binding SIGN-R1 expressed on sensor marginal zone splenic macrophages in mice [18], [20], [35]. Results from our studies of IVIG protection against fatal HSE induced by dysregulated CNS inflammation not only support, but extend the model by demonstrating existence of a second pathway induced by the non-sIgG fraction that was highly effective even with low dose IVIG. Whereas FcγRIIb was essential for prevention of ITP and arthritis by sIgG, it was not important for protection against fatal HSE by low dose IVIG (i.e., lacking sIgG). The non-sIgG pathway induced Tregs as well as ICOS^+^ CD4^+^ T cells that produced IL-10, the latter being essential for the anti-inflammatory effects of low dose IVIG. However, Kaneko et. al. reported that desialylated IVIG failed to protect against serum induced arthritis, as inflammatory infiltration of the joints was not inhibited [21]. This discrepancy may be due to differences in the experimental models used. Alternatively, the non-sIgG fraction of IVIG may function optimally to elicit anti-inflammatory responses in the context of virus induced inflammatory diseases as opposed to autoimmune diseases. In sum, our results significantly advance understanding of IVIG's anti-inflammatory and immunomodulatory activities and reveal the potential utility of IVIG for treating viral infections in which excessive inflammatory responses contribute to disease pathology, such as in West Nile virus and highly pathogenic influenza virus infections.

## Materials and Methods

### Ethics Statement

This study was carried out in strict accordance with the recommendations in the *Guide for the Care and Use of Laboratory Animals* of the National Institutes of Health. All animal studies were conducted under a protocol approved by the Institutional Animal Care and Use Committee (IACUC, Permit # A3001-01) of City of Hope to ensure the highest ethical and humane standards were followed.

### Mice and Virus Inoculation

129S6 WT (Taconic, Hudson, NY), 129 FoxP3-GFP [53] and 129 IL-10 KO (Jackson Laboratories, Bar Harbor, Maine) mice were bred in the vivarium at City of Hope. Male mice of 6–8 weeks of age were infected with HSV 17+ strain. Mice were sedated with ketamine (60 mg/kg) and xylazine (5 mg/kg) prior to HSV inoculation by corneal scarification with 3200 PFU (equivalent to 10 LD_50_ for the 129 WT strain) as previously described [3]. Infected mice were monitored daily as previously described [3].

### Administration of Intravenous Immunoglobulin (IVIG) and Antibodies

Pooled human serum was obtained from Sigma-Aldrich (St. Louis, MO, USA) and IVIG (Carimmune, NF) was obtained from CSL Behring (King of Prussia, PA, USA). The recipient group was intraperitoneally (i.p.) injected with 0.5 ml of either human sera or IVIG (3.75 mg/mouse, as indicated) 24 h pi. IVIG and various derivatives were administered 24 h pi unless otherwise stated. The dose of IVIG was 3.75 mg/mouse, based on an earlier study [9]. IVIG dose ranging studies demonstrated that 1.5 mg was the minimal protective dose for HSV infected 129 mice (**Figure S1**).

For some experiments, HSV antibodies were removed from IVIG by adsorption with HSV infected monolayers of Vero cells. Adsorbed antibody was used after confirming the absence of neutralizing activity [8]. Desialylation and deglycosylation of IVIG was performed as described previously [32]. Briefly, for desialylation, IVIG (100 mg) in sodium citrate buffer (0.05 M, pH 6.0) was incubated (37 C, 40 h) with 700 units of recombinant *α*2,3 or *α*2,3/*α*2,6 neuraminidase (*Clostridium perfingens*, New England BioLabs). For deglycosylation, IVIG (100 mg) in sodium phosphate buffer (0.2 M, pH 8.5) was incubated (37°C, 48 h) with 25,000 units of PNGaseF (*Flavobacterium meningosepticum*, New England BioLabs). Monomer fractions of deglycosylated or desialylated IVIG preparation (3 mg/mouse) purified by HPLC were used. For depletion of FoxP3^+^ CD4^+^ Tregs and FcγRIIb/II expressing CD11b^+^ cells, mice were injected with 4 doses of either anti-CD25 mAb PC61 (250 µg) or anti-FcγRIIb/III mAb 2.4G2 (500 µg) on days −2, 0, +1, +2 pi. All mice were inoculated with HSV at day 0 pi and received either IVIG or PBS i.p. on day +1 pi. Depletion of cell subsets was confirmed by flow cytometry.

### Isolation of Mononuclear Cells from the CNS

CNS derived mononuclear cells were isolated as previously described [3]. Briefly, brains, BS and spinal cords were removed separately from mice perfused with PBS, minced and digested with collagenase and DNAse for 30 min prior to centrifugation (1250× g, 50 min) on a two step Percol gradient [3]. Brain refers to the whole brain minus the brainstem. The enriched population contained CNS infiltrating CD45^high^ cells, CD45^int^ microglia and CD45^neg^ CNS resident glial cells. CD45^high^ cells comprised ∼ 5–8% of total mononuclear cells isolated from the BS of naïve 129 WT mice. Cell viability was greater than 95% as revealed by trypan blue staining. We confirmed that enzyme digestion did not affect expression of cell surface markers.

### Flow Cytometric Analysis

Single cell suspensions isolated from either, brain, BS, spleen or CLN were blocked with a 10% Fmixture of normal mouse, rat and horse serum and rat anti-mouse CD16/32 (2.4G2, BD PharMingen) for 15 min prior to incubation with antibodies (Abs) to determine cell surface expression of various markers. Phycoerythrin (PE), FITC or allophycocyanin conjugated Abs specific for F480 (BM8), CD11b (M1/70), Gr-1 (RB6-865), CD8 (53-6.7), CD4 (RM4-4), ICOS (7E.17G9), CD62L (MEL-14), CD25 (PC61.5), IFN-γ (XMG1.2), IL-10 (JES5-16E3), FcεR1 (MAR-1), MHC class I (28-14-8) and class II (M5/114-15.2) were obtained from eBioscience (San Diego, CA). FITC, PE or PerCP conjugated Ly6-G (1A8), Ly6-C (AL-21), CD45 PerCP (30-F11), CD107a (1D4B) and CD107b (ABL-93) were obtained from BD PharMingen (San Jose, CA). All isotype controls were obtained from eBioscience. In the Figure Legends and Results, all references to infiltrating cells or inflammatory cells from either BS or brain refer to mononuclear cells isolated from either compartment distinguished by their CD45^high^ expression. F480^+^ macrophages were characteristically CD45^high^, CNS resident microglia CD45^int^ and glial cells CD45^neg^. Activation of both cell subsets was determined by their mean fluorescence intensity of expression of MHC class II molecules. Efficiency of degranulation by macrophages was determined *in vitro* in the absence of antigen stimulation or following stimulation of cells for 5 h with heat-killed HSV in the presence of anti-CD107a/b antibodies to capture cell surface associated LAMPs. Resting macrophages did not express surface CD107a (data not shown). Neutrophils were determined by their SSC^high^, Gr-1^high^, Ly6-G^+^, MHC II^−^, F480^−^ phenotype. CD4^+^ Tregs were determined by reactivity to CD25 and FoxP3 GFP expression in the 129 FoxP3 GFP reporter mice. Cells were acquired on a Cyan ADP Analyzer (Beckman Coulter, Fullerton CA) and flow cytometry analysis was performed using Flowjo software (Treestar Inc.). In Results, where percentage of cell subsets is calculated within specific populations, the specific population is gated and considered to be 100%, while cell subsets are expressed as a fraction thereof. For example, in Figure 2E, the top left plot shows BS mononuclear cells isolated from IVIG-treated HSV-infected mice at d6 pi. The CD45^high^ infiltrating cells comprise 6.6% of total mononuclear cells. CD11b^+^ monocytes therefore comprise 55% (3.6% CD11b^+^ ÷ 6.6% CD45^high^) of total CD45^high^ infiltrating cells within the BS.

### Determination of BBB Integrity

To determine the integrity of the BBB after HSV corneal infection, mice were injected i.p. with 10% sodium fluorescein (Sigma Aldrich, PA). After 10 min, mice were bled and perfused and the BS, brain and spinal cord collected and frozen on dry ice. The organs were weighed and homogenized in 10% w/vol PBS. Homogenates were treated with 15% TCA and extracted with 1.5 M NaOH. The supernatants were analyzed for fluorescence at 475 nm, and the amount of sodium fluorescein in sera and BS were extrapolated from a standard curve. The following formula was used to calculate the amount of sodium fluorescein in BS or brain: (mg fluorescein in brain tissue/mg of protein)/(mg fluorescein in sera/ml of blood) and the result was expressed as fold increase in fluorescence in comparison to naïve mice [54].

### Preparation and Purification of F(ab)_2_ and Fc Fragments, Monomeric and Dimeric IVIG and Cu^64^-Labeled Monomeric IVIG

To prepare F(ab)_2_ and Fc fragments, IVIG was dialyzed into PBS using a Mini Dialyzer cassette (10 kDa, Thermo Scientific, Rockford, IL) prior to enzymatic cleavage with either Pepsin or papain (Thermo Scientific) using the manufacturer's protocol. The reaction was stopped by spinning down the enzyme-linked beads and the reaction mixture was dialyzed into PBS prior to partitioning the Fc and F(ab)_2_ fragments using HPLC. The F(ab)_2_ fragments retained antigen binding and neutralizing activities. Aggregated and non-aggregated IgGs were separated using HPLC, and these comprised primarily head-to-head dimers and monomers of IgG, respectively. The non-aggregated fraction of IVIG was collected based on size and the purity determined by SDS PAGE electrophoresis. To label IVIG with radiolabeled ^64^Cu, IVIG was conjugated with DO3A-VS (1,4,7-tris(carboxymethyl)-10-(vinylsulfone)-1,4,7,10-tetraazacyclododecane), a bifunctional chelator, under Argon for 2 h at room temperature as previously described [55]. Labeling with ^64^Cu was performed in 0.1 M ammonium citrate, pH 5.5 for 45 min at 43°C and the reaction terminated by addition of 10 mM DTPA to achieve a final concentration of 1 mM. The radiolabeled product was purified on a size exclusion column. HSV infected or naïve mice were injected with IVIG spiked with 50 ul of ^64^Cu-labeled IVIG (50 µl) at 24 h pi. At 4–8 h intervals over a 48 h period, mice were imaged with a small animal PET scanner (microPET R4, Siemens/CTIMI, Knoxville, TN). At 44–48 h pi, animals were euthanized and organs such as spleen, liver, brain, BS, trigeminal ganglia, etc. were weighed and assayed for radioactivity using a gamma counter. All image processing and analysis was performed using standard microPET software [55].

### Statistical Analysis

Graph Pad Prizm Software was used to analyze mortality data by log rank (Mantel Cox) test, taking into account both time of death and mortality.

## Supporting Information

Figure S1 Effect of dose and time of IVIG administration on protection. **(A)** 129 WT mice infected with HSV 17+ by corneal scarification were given 3.75 mg IVIG i.p. at indicated times pi or **(B)** different doses of IVIG at 24 h pi.
(TIF)

Figure S2 SNA lectin blotting to detect sIgG. S^+^ IgG and S^−^ IgG were purified from IVIG and HSV seronegative pooled serum by affinity chromatography on SNA lectin columns and blotted for reactivity to SNA lectin (top) or anti-human IgG (bottom).
(TIF)

Figure S3 Bio-distribution of ^64^Cu-labeled IVIG in HSV infected 129 WT mice and naïve mice. Mice injected i.p. with purified ^64^Cu-labeled IgG at 24 h pi were sacrificed at 44 h after IgG infusion, various tissues were dissected from infected and naïve mice and radioactivity was determined by gamma counting. The average percent injected activity dose per gram organ for uninfected and infected mice was calculated after correcting for radio-decay.
(TIF)

Figure S4 CD45^high^ cells infiltrating the spinal cords of HSV infected mice. CD45^high^ infiltrates (determined by flow cytometry) in mice treated with IVIG or PBS at d6 and d8 pi.
(TIF)

Figure S5 Intracellular staining of BS infiltrating cells for IL-10 and IFN-γ. Mononuclear cells isolated at d6 pi **(A)** or d14 pi **(B)** from BS of HSV infected mice given PBS or IVIG were stimulated with (blue dots) or without (red) PMA + ionomycin and analyzed for intracellular IFN-γand IL-10 by flow cytometry. Percentages in top row indicate percent of cells positive for cytokine expression within CD45^high^ subset.
(TIF)
