# Supplementary figures and images for: Passively Administered Pooled Human Immunoglobulins Exert IL-10 Dependent Anti-Inflammatory Effects that Protect against Fatal HSV Encephalitis
Source: PLoS Pathog. 2011 Jun 2;7(6):e1002071. doi: 10.1371/journal.ppat.1002071 (PMC3107211; doi:10.1371/journal.ppat.1002071)

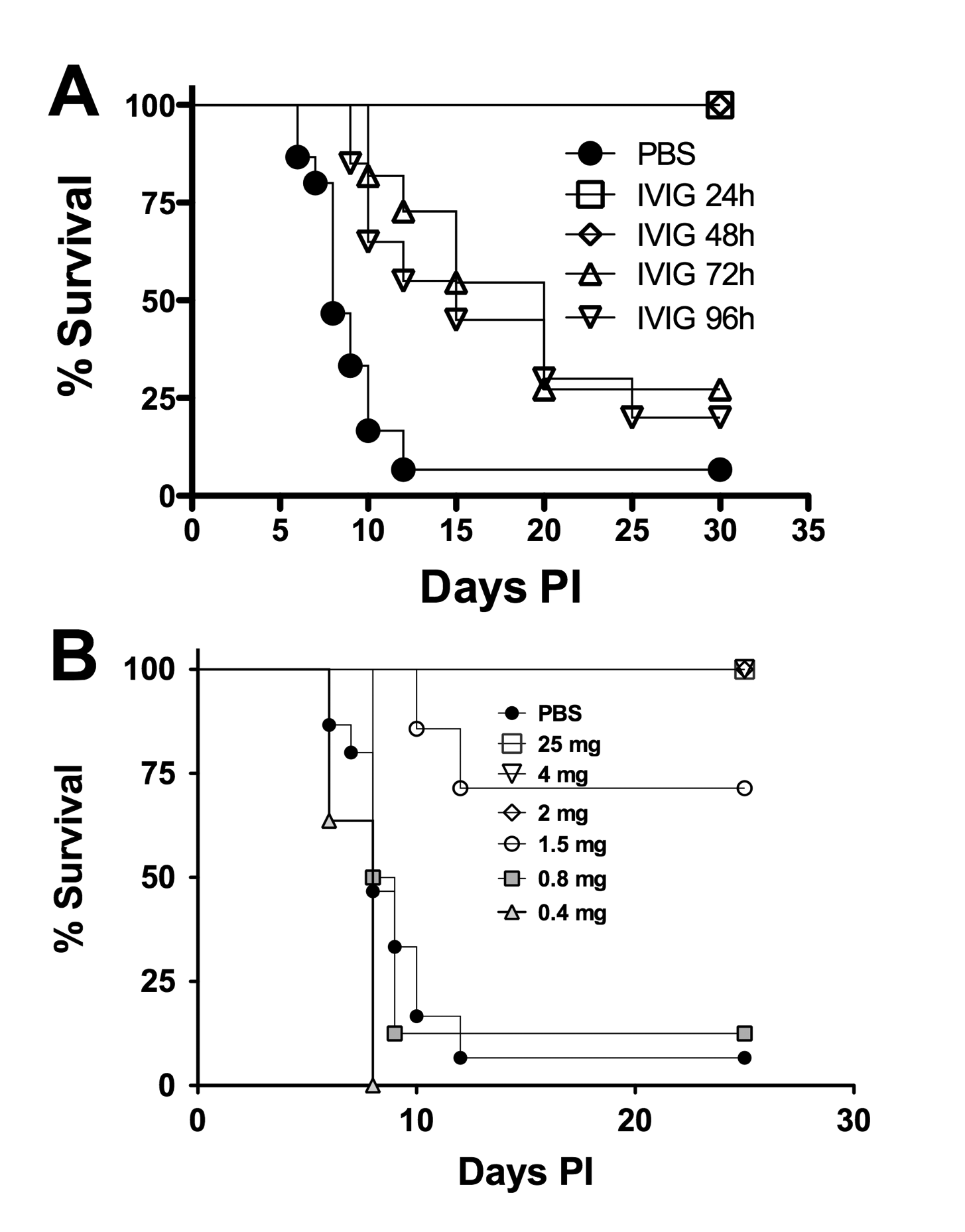

Supplement: Figure S1 — Effect of dose and time of IVIG administration on protection. (A) 129 WT mice infected with HSV 17+ by corneal scarification were given 3.75 mg IVIG i.p. at indicated times pi or (B) different doses of IVIG at 24 h pi. (TIF) [file ppat.1002071.s001.tif]

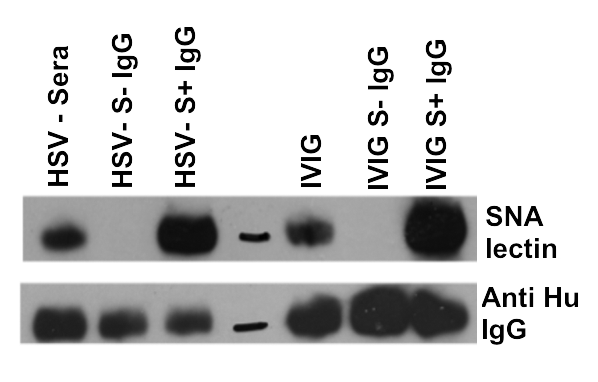

Supplement: Figure S2 — SNA lectin blotting to detect sIgG. S+ IgG and S− IgG were purified from IVIG and HSV seronegative pooled serum by affinity chromatography on SNA lectin columns and blotted for reactivity to SNA lectin (top) or anti-human IgG (bottom). (TIF) [file ppat.1002071.s002.tif]

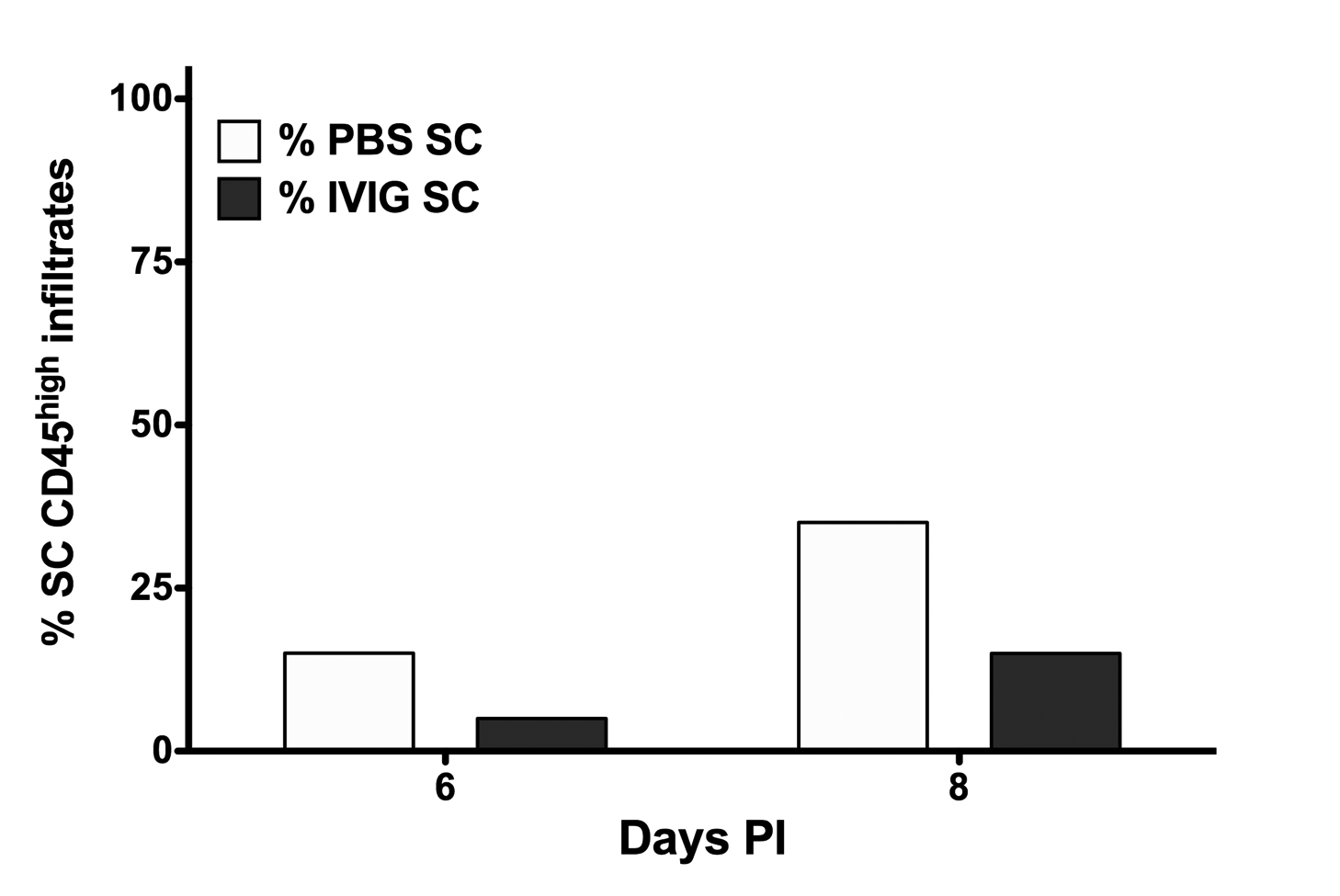

Supplement: Figure S3 — Bio-distribution of 64Cu-labeled IVIG in HSV infected 129 WT mice and naïve mice. Mice injected i.p. with purified 64Cu-labeled IgG at 24 h pi were sacrificed at 44 h after IgG infusion, various tissues were dissected from infected and naïve mice and radioactivity was determined by gamma counting. The average percent injected activity dose per gram organ for uninfected and infected mice was calculated after correcting for radio-decay. (TIF) [file ppat.1002071.s003.tif]

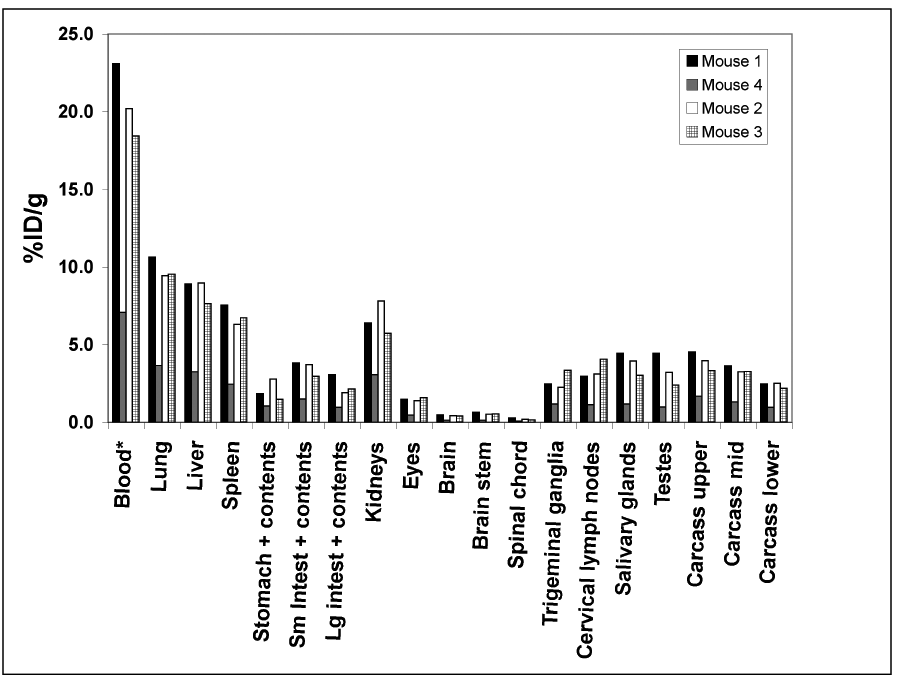

Supplement: Figure S4 — CD45high cells infiltrating the spinal cords of HSV infected mice. CD45high infiltrates (determined by flow cytometry) in mice treated with IVIG or PBS at d6 and d8 pi. (TIF) [file ppat.1002071.s004.tif]

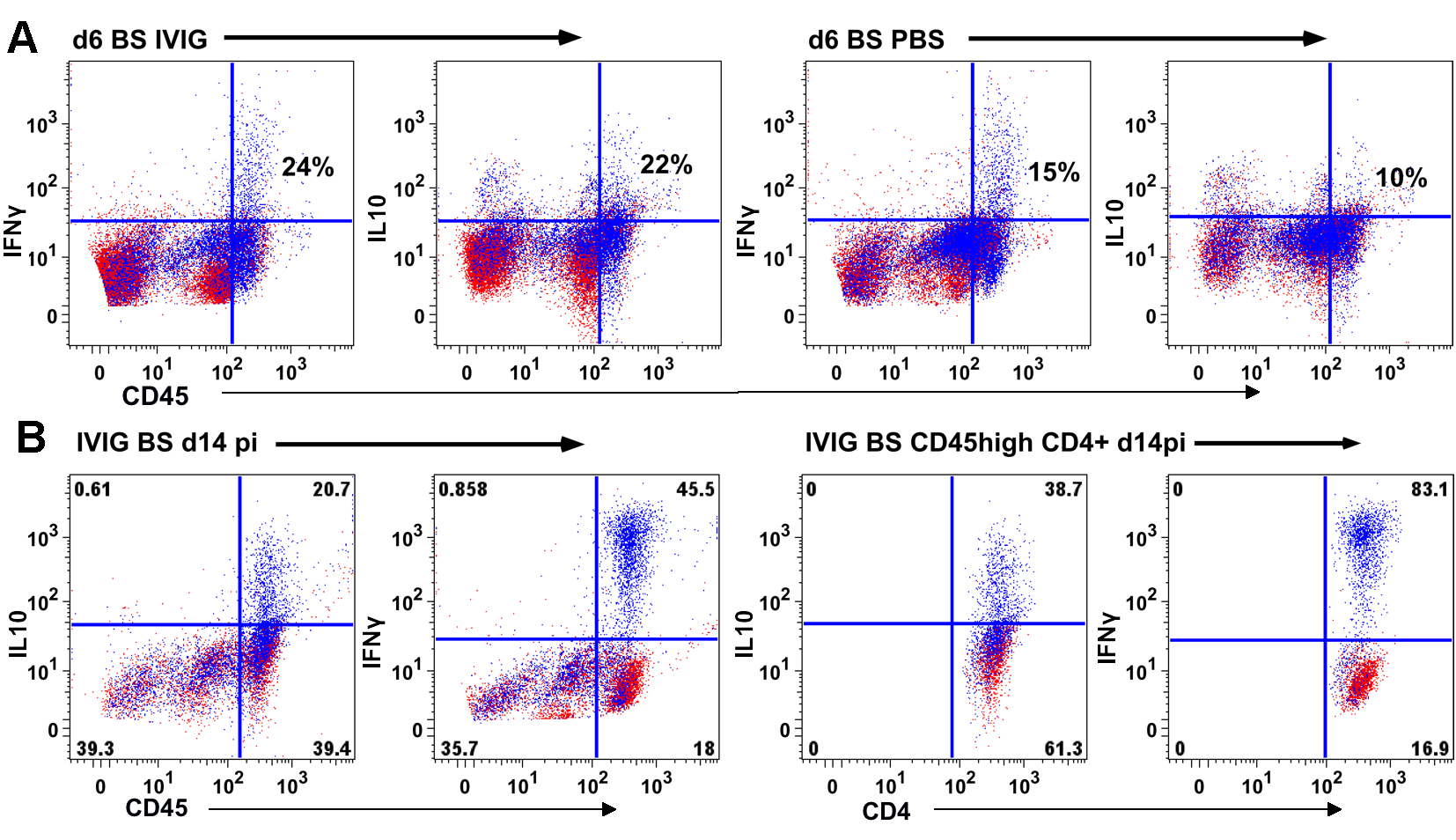

Supplement: Figure S5 — Intracellular staining of BS infiltrating cells for IL-10 and IFN-γ. Mononuclear cells isolated at d6 pi (A) or d14 pi (B) from BS of HSV infected mice given PBS or IVIG were stimulated with (blue dots) or without (red) PMA + ionomycin and analyzed for intracellular IFN-γand IL-10 by flow cytometry. Percentages in top row indicate percent of cells positive for cytokine expression within CD45high subset. (TIF) [file ppat.1002071.s005.tif]
